# Optimized K^+^ Deposition Dynamics via Potassiphilic Porous Interconnected Mediators Coordinated by Single‐Atom Iron for Dendrite‐Free Potassium Metal Batteries

**DOI:** 10.1002/advs.202413804

**Published:** 2025-01-09

**Authors:** Tzu‐Chi Lin, Yi‐Chun Yang, Hsing‐Yu Tuan

**Affiliations:** ^1^ Department of Chemical Engineering National Tsing Hua University Hsinchu 30013 Taiwan

**Keywords:** hierarchical porous carbon, potassiphilic sites, potassium metal batteries, Scharifker‐Hills, single atom

## Abstract

Potassium metal batteries are emerging as a promising high‐energy density storage solution, valued for their cost‐effectiveness and low electrochemical potential. However, understanding the role of potassiphilic sites in nucleation and growth remains challenging. This study introduces a single‐atom iron, coordinated by nitrogen atoms in a 3D hierarchical porous carbon fiber (Fe─N‐PCF), which enhances ion and electron transport, improves nucleation and diffusion kinetics, and reduces energy barriers for potassium deposition. Molten potassium infusion experiments confirm the Fe─N‐PCF's strong potassiphilic properties, accelerating adsorption kinetics and improving potassium deposition performance. According to the Scharifker‐Hills model, traditional carbon fiber substrates without potassiphilic sites cause 3D instantaneous nucleation, leading to dendritic growth. In contrast, the integration of single‐atom and hierarchical porosity promotes uniform 3D progressive nucleation, leading to dense metal deposition, as confirmed by dimensionless i^2^/i_max_^2^ versus t/t_max_ plots and real‐time in situ optical microscopy. Consequently, in situ X‐ray diffraction demonstrated stable potassium cycling for over 1900 h, while the Fe─N‐PCF@K||PTCDA full cell retained 69.7% of its capacity after 2000 cycles (72 mAh g^−1^), with a low voltage hysteresis of 0.876 V, confirming its strong potential for high energy density and extended cycle life, paving the way for future advancements in energy storage technology.

## Graphical abstract

A novel strategy enhances potassium metal batteries (PMBs) by single‐atom Fe coordinated within N‐doped hierarchical porous carbon fibers (Fe─N‐PCF), promoting uniform K^+^ nucleation, efficient charge transfer, and stable, dendrite‐free deposition.

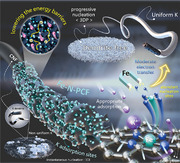

## 1 Introduction

Potassium (K) metal holds promise for next‐generation high‐energy batteries due to its abundance (1.5 wt.%), low redox potential (−2.93 V vs SHE), and high specific capacity (687 mA h g⁻^1^).^[^ ^1^, ^2^, ^3^ ^]^ However, uneven deposition and volume expansion cause solid electrolyte interphase (SEI) degradation, dendrite formation, and capacity loss, posing safety risks.^[^ ^4^, ^5^, ^6^ ^]^ While conventional strategies like electrolyte optimization and artificial SEI layers stabilize the SEI, they fail to prevent dendrite growth and volume changes. 3D conductive scaffolds offer potential for dendrite‐free potassium metal anodes (PMAs),^[^ ^7^, ^8^, ^9^ ^]^ but their poor K affinity limits controlled deposition. Introducing potassiphilic (referring to a material's strong affinity or attraction to potassium ions (K^+^)) heteroatoms, such as sulfur‐modified Ti_3_C_2_‐chitosan aerogels, can reduce nucleation barriers and promote uniform deposition, though the underlying mechanisms remain unclear.^[^ ^10^ ^]^ Advancing dendrite‐free, long‐lasting PMAs with improved cycling stability and safety requires addressing this gap.

To achieve dendrite‐free PMAs, host materials must exhibit high binding energy, low nucleation and diffusion barriers, good wettability, rapid nucleation kinetics, and ample nucleation sites.^[^ ^11^ ^]^ These factors synergistically ensure uniform K deposition. Specifically, Young's equation links the contact angle (referring to the angle formed between a liquid droplet and the surface of a solid material at the point) to Gibbs free energy, with changes in the contact angle expressed as:^[^ ^12^ ^]^

(1) $$\cos\theta =\frac{\gamma_{S-E}-\gamma_{S-K}}{\gamma_{K-E}}$$

where γ(S‐E), γ(S‐K), and γ(K‐E) represent the interfacial energies between the substrate‐electrolyte, substrate‐K metal, and K metal‐electrolyte, respectively. Enhancing the binding energy between the substrate and K metal reduces γ(S‐K), lowers the contact angle, and minimizes K nucleation volume, promoting a lower initial deposition density. A heteroatom‐doped carbon framework (P‐PMCF) offers high binding energy and potassiphilic sites, reducing critical nucleation size and preventing dendrite formation. According to classical nucleation theory, the heterogeneous nucleation barrier is inversely related to the contact angle, described by the following equation:^[^ ^13^ ^]^

(2) $$\Delta {G_{\mathrm{het}\;}}^{*}=f\left(\theta \right)\Delta {G_{\mathrm{hom}\;}}^{*}$$

where ΔG_het_^*^ and ΔG_hom_^*^ represent the heterogeneous and homogeneous nucleation barriers, respectively. The function f(θ) decreases as the contact angle (θ) decreases, indicating that a smaller wetting angle corresponds to a lower nucleation energy barrier. Lowering the self‐diffusion energy barrier has been shown to enhance atom diffusion, which effectively suppresses dendrite growth. Wettability of molten K on the substrate, governed by atomic and molecular energy interactions, impacts charge transfer at the interface. As molten K infiltrates the porous substrate, capillary force, described by the Young‐Laplace equation:^[^ ^14^ ^]^

(3) $$\Delta P=\frac{2\sigma \cos\theta }{r}$$

is influenced by surface tension (σ), droplet curvature (r), and contact angle (θ). Surface modification enhances binding energy, increasing cos(θ) and thereby strengthening capillary forces, improving substrate wettability, contact with K, charge transfer stability, local resistance, and current density uniformity. Functionalizing carbon scaffolds with amine groups transforms them from non‐wettable to super‐wettable, enabling rapid molten K infiltration and forming a uniform carbon‐K composite anode. According to classical nucleation theory, thermodynamic properties regulate K‐substrate interactions, while the Scharifker‐Hills (SH) model outlines the nucleation rate and growth kinetics.^[^ ^15^ ^]^

(4) $$N=N_{0}\left[1-\exp\left(-At\right)\right]$$

within a specified time (t), N denotes the density of nucleation sites on the substrate, while N_0_ represents the density of available nucleation sites, and A indicates the nucleation rate. When A significantly exceeds 1/t, rapid nucleation occurs, but limited nucleation sites cause localized deposition and accelerate dendrite growth. A higher density of nucleation sites promotes uniform deposition, enabling continuous progressive nucleation and preventing dendrite formation. Adding an ion‐conductive hydroxyapatite interface on zinc surfaces may shift electroplating from instantaneous to high‐speed progressive nucleation, producing small, dense crystals and reducing dendritic growth. Enhancing PMAs through heteroatom incorporation, functional group modification, and SEI optimization has improved cycling performance, but a systematic integration of these strategies is crucial for further anode optimization.

Recently, single‐atom materials (SAMs) have become a key focus in materials science. By incorporating isolated metal atoms on conductive substrates to form MNC sites (metal, nitrogen (N), and carbon (C)), SAMs enhance delocalized electrons, boosting electronic conductivity and electrochemical performance.^[^ ^16^, ^17^ ^]^ Following the success of the single‐atom Pt/FeO_x_ catalyst, research on SAM properties and catalytic mechanisms has surged.^[^ ^18^ ^]^ SAMs offer ≈100% atomic utilization, with metal‐support interactions altering charge transfer and electron distribution, ensuring stability in harsh conditions. These features make SAMs highly effective in photocatalysis, electrocatalysis, and energy applications. For instance, Jiang et al. synthesized a Pt‐CuO catalyst that modulated electronic metal‐support interaction (EMSI), significantly enhancing acetone oxidation.^[^ ^19^ ^]^ Using porous materials as supports increases active site exposure, improving catalytic efficiency and preventing metal aggregation. Hierarchical porous structures further enhance performance by facilitating reactant diffusion.^[^ ^20^ ^]^ Jung et al. developed cobalt‐rich single‐walled carbon nanohorns, which formed a Co‐N_4_ configuration after annealing, boosting both hierarchical porosity and electrocatalytic activity for the oxygen reduction reaction.^[^ ^21^ ^]^

**Scheme 1:**
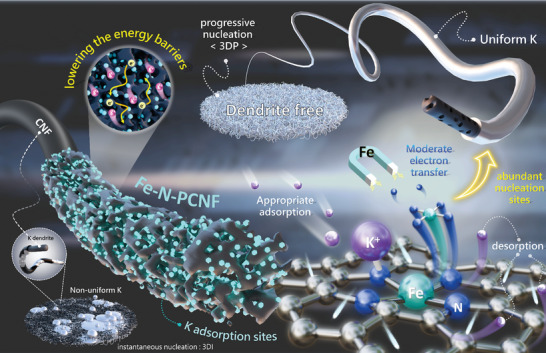
Schematic illustration of Fe single atoms embedded in hierarchical porous carbon fibers. The abundance of single atomic sites significantly accelerates nucleation and diffusion kinetics by serving as potassiphilic sites, optimizing charge transfer and enhancing the reversibility of potassium metal adsorption/desorption. Simultaneously, these numerous nucleation sites regulate the growth behavior, transitioning into a progressive nucleation mode, ultimately achieving a dendrite‐free PMA.

This study integrates multiple theoretical and material design approaches to optimize K metal deposition and prevent dendrite formation. We propose a novel iron‐centered single‐atom structure (Fe‐N_4_) that enhances charge density distribution via EMSI, improving electron transfer and facilitating K^+^ absorption while reducing nucleation and diffusion barriers (**Scheme** **1**). The Fe‐N_4_ structure in Fe‐N doped porous carbon nanofibers (Fe‐N‐PCF) increases adsorption energy with K, enhancing wettability and guiding uniform nucleation. This atomically dispersed structure provides abundant nucleation sites, promoting dense, dendrite‐free K deposition consistent with the SH model for 3D progressive nucleation. Strong Fe‐N covalent bonds ensure thermodynamic stability and structural integrity during cycling. The Fe‐N_4_ sites in N‐doped porous carbon nanofibers (N‐PCF) create a self‐supporting structure with a large surface area, reducing localized current density, improving electron and ion transport, and accommodating volume expansion, leading to excellent rate performance and long‐term cycling stability. In summary, the dual strategy of Fe‐N_4_ single‐atom configurations and hierarchical porous structures significantly enhances K metal nucleation and diffusion, offering a promising solution for PMA.

## 2 Results and Discussion

**Figure 1:**
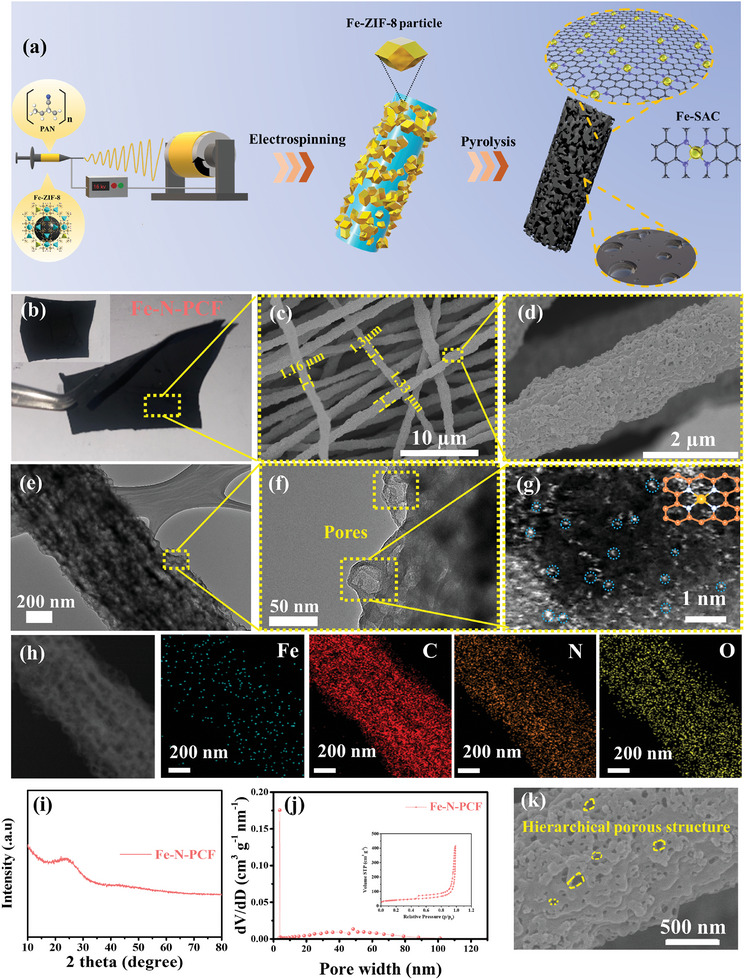
a) Schematic illustration of the preparation process of Fe‐N‐PCF. b) Digital photo of Fe‐N‐PCF and the inset illustrates its flexibility. c,d) SEM images of Fe‐N‐PCF at various magnifications. e,f) TEM image, g) HAADF‐STEM image, and h) corresponding STEM element mapping images of Fe‐N‐PCF. i) XRD pattern, j) nitrogen adsorption‐desorption isotherms and the corresponding pore width distribution curves for Fe‐N‐PCF and k) corresponding magnified SEM image.

Fe‐N‐PCF features a hierarchically porous hollow structure with abundant single‐atom active sites supported by a 3D framework, optimizing electrolyte and electron transport. The synthesis utilizes Zn‐based ZIF‐8, where Zn^2+^ coordinates with 2‐methylimidazole ligands (**Figure** **1**a). Due to the strong affinity between Fe and N, Fe replaces Zn in competitive coordination environments, forming stable bonds and uniformly incorporating Fe atoms into the carbon matrix. As a result, Fe─ZIF‐8 nanoparticles serve as carriers for Fe atoms. X‐ray diffraction (XRD) analysis confirms the structural integrity of ZIF‐8 after Fe substitution (Figure S1, Supporting Information). Scanning electron microscopy (SEM) image reveals that the synthesized crystals exhibit the characteristic rhombic dodecahedron morphology of ZIF‐8, with an approximate particle size of 400 nm (Figure S2, Supporting Information). During high‐temperature pyrolysis, Zn evaporates, forming micropores that spatially isolate metal atoms but impede mass and ion transport. To overcome the limitations of the microporous structure, Fe─ZIF‐8 and PAN were first dissolved in DMF to be electrospun and carbonized, yielding mesoporous carbon cages within interconnected nanofibers, referred to as Fe‐N‐PCF.

As shown in Figure 1b, the synthesized Fe‐N‐PCF demonstrates excellent flexibility and mechanical stability, retaining its original morphology without breaking during bending and folding, thanks to the interconnected network of electrospun Fe─ZIF‐8/PAN nanofibers. After thermal treatment in N_2_ gas, SEM images (Figure 1c) reveal that Fe‐N‐PCF, with an average diameter of 1.25 ± 0.1 µm, forms a loosely aggregated 3D network of randomly intertwined carbon fibers.^[^ ^22^ ^]^ High‐magnification SEM analysis of Fe‐N‐PCF confirms its rough surface with uniformly distributed pores in the carbon fibers (Figure 1d), along with continuous conductive pathways. This indicates that the confined carbonization within the PAN matrix prevents aggregation of carbonized Fe─ZIF‐8 nanoparticles. The addition of Fe─ZIF‐8 increases the roughness and porosity of the carbon fibers. Both N‐PCF and Fe‐N‐PCF show similar surface characteristics and morphologies (Figure S3a,b, Supporting Information), confirming that single‐atom doping does not impact ZIF‐8 contraction during pyrolysis. In contrast, CF displays a smooth and continuous fiber structure (Figure S4a,b, Supporting Information).

Transmission electron microscopy (TEM) images of Fe‐N‐PCF (Figure 1e) reveal densely packed nanopores within carbon fibers, forming a hollow interconnected framework. This porous structure increases active site exposure, enhances electrolyte contact, and improves K^+^ diffusion and electron transport. High‐resolution TEM (HRTEM) images show hollow features smaller than 50 nm, caused by the contraction of Fe─ZIF‐8 (Figure 1f). High‐angle annular dark‐field scanning transmission electron microscopy (HAADF‐STEM), helps in observing the distribution of elements, atomic arrangements, and structural features with high resolution, reveals no Fe nanoparticles, only highly dispersed bright spots corresponding to isolated Fe single atoms (Figure 1g). The enhanced contrast of these spots, due to Fe's higher atomic number compared to N, C, and oxygen (O), confirms the atomic‐scale dispersion of Fe^2+^ within the ZIF‐8‐derived structure. Energy‐dispersive X‐ray (EDX) spectroscopy shows uniform distribution of Fe, N, C, and O across the carbon fibers, further verifying the homogeneous dispersion of Fe single atoms and effective N doping (Figure 1h). The XRD pattern of Fe‐N‐PCF shows no crystalline metallic phases, indicating no metal aggregation during pyrolysis (Figure 1i). This confirms that ZIF‐8′s spatial confinement stabilizes Fe atoms at the atomic scale. Two broad diffraction peaks at 24° and 43°, corresponding to the (002) and (100) planes of amorphous graphitic carbon,^[^ ^23^ ^]^ suggest low crystallinity and high structural defects in the carbon matrix, likely due to heteroatom incorporation. Similarly, XRD patterns of N‐PCF and N‐CF show broad peaks, confirming the amorphous nature of the electrospun fibers after pyrolysis (Figures S5 and S6, Supporting Information).

Nitrogen adsorption‐desorption analysis was used to evaluate the pore size distribution and specific surface area (SSA) of Fe‐N‐PCF (Figure 1j). The isotherms show a typical type IV curve, indicating mesoporous characteristics. At low relative pressures (P/P₀ < 0.01), adsorption corresponds to micropores, likely formed by Zn evaporation during pyrolysis. In the intermediate and high‐pressure regions (P/P₀ = 0.44–0.99), the hysteresis loop and increasing adsorption confirm the presence of micropores, mesopores, and macropores (Figure 1k). Mesopores arise from Fe─ZIF‐8 transforming into hollow nanocages that merge into macropores, creating a hierarchical porous structure that exposes single Fe atoms and boosts catalytic activity. The combination of Fe─ZIF‐8 and N‐doped PAN during pyrolysis results in high SSA and a multifunctional pore structure, enhancing electrochemical surface areas and charge/mass transfer. N_2_ isotherms for N‐PCF also show a type IV curve (Figure S7, Supporting Information), indicating a similar hierarchical structure, while carbon nanofibers (CF) exhibit a reduced surface area due to its lack of porosity (Figure S8, Supporting Information).

**Figure 2:**
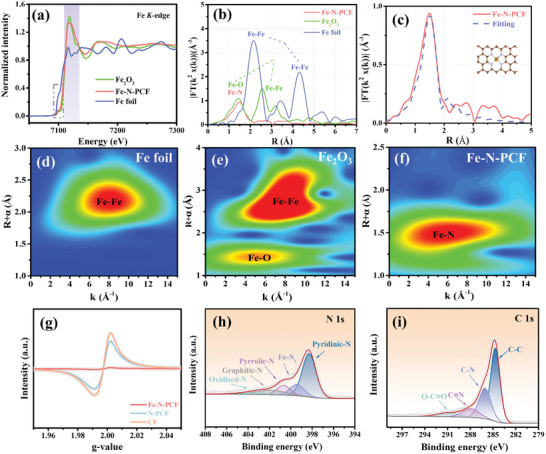
a) XAS spectra and b) Fourier‐transform EXAFS spectra in R‐space of Fe‐N‐PCF, Fe foil, and Fe_2_O_3_, and c) corresponding FT‐EXAFS fitting of Fe K‐edge in R‐space of Fe‐N‐PCF and FePc. Wavelet transform contour plot of the d) Fe foil, e) Fe_2_O_3_, and f) Fe‐N‐PCF k^2^‐weighted EXAFS signals. g) EPR spectrum of Fe‐N‐PCF, N‐PCF, and CF. High‐resolution XPS spectrum of h) N 1s and i) C 1s for Fe‐N‐PCF.

Optimizing structural configurations and active site arrangements significantly impacts electrochemical performance. Fe K‐edge X‐ray absorption spectroscopy (XAS) was used to examine the chemical states of Fe sites in Fe‐N‐PCF, with Fe foil and Fe_2_O_3_ as references. The XAS spectrum shows the absorption threshold of Fe‐N‐PCF between those of Fe foil and Fe_2_O_3_, suggesting Fe atoms are in a +3 oxidation state (**Figure** **2**a).^[^ ^24^ ^]^ A pre‐edge peak near 7115 eV, corresponding to the 1s → 4p_x_ transition, indicates a square planar Fe‐N_4_ configuration. The pre‐edge feature in Fe_2_O_3_ is attributed to its octahedral C3v symmetry, though Fe_2_O_3_ does not form during high‐temperature pyrolysis in a carbon‐rich N_2_ inert environment.^[^ ^25^ ^]^ A Fourier‐transform k^2^‐weighted extended X‐ray absorption fine structure (EXAFS) analysis further clarified the coordination structure (Figure 2b). The Fe_2_O_3_ spectrum shows a Fe─O peak at 1.44 Å, while Fe‐N‐PCF exhibits a shifted peak at 1.49 Å for Fe‐N bonds.^[^ ^26^ ^]^ Fe_2_O_3_ also has a Fe─Fe peak at 2.6 Å. In contrast, Fe foil shows characteristic Fe─Fe peaks at 2.2 and 4.4 Å. However, Fe‐N‐PCF only displays a peak at 1.5 Å for Fe‐N bonds, with no Fe─Fe or Fe─O peaks, indicating isolated Fe single atoms forming strong Fe‐N bonds. This agrees with previous HAADF‐STEM and EDX analyses, confirming the dispersion of Fe atoms within the carbon fiber. To precisely characterize the coordination environment of Fe atomic sites, EXAFS fitting analysis was performed on the first coordination shell of Fe‐N‐PCF. The fitting results show a strong match with the theoretical model (Figure 2c and Table S1, Supporting Information), confirming that each Fe atom is coordinated to four N atoms, forming the Fe‐N_4_ configuration within the carbon fiber.^[^ ^27^, ^28^ ^]^ This Fe‐N_4_ structure provides potassiphilic sites, reducing K nucleation overpotential in potassium metal batteries (PMBs). In R‐space, the Fe‐N bond length remains ≈2 Å, indicating the Fe‐N_4_ planar geometry is retained after doping (inset, Figure 2c). Wavelet transform (WT) analysis was performed to further clarify the coordination environment of the central Fe atom (Figure 2d–f). WT in both k‐space and R‐space enables high‐resolution differentiation of backscattering atoms around the Fe site. The WT plot of Fe_2_O_3_ shows a local maximum at 5.1 Å⁻^1^ for the Fe─O scattering pathway, while Fe‐N‐PCF exhibits a distinct Fe‐N pathway at 6.1 Å⁻^1^. In Fe‐N‐PCF, intense peaks correspond to Fe‐N coordination, whereas Fe foil and Fe_2_O_3_ show a Fe─Fe peak at 8.1 Å⁻^1^, absent in Fe‐N‐PCF. This confirms that Fe atoms are isolated and uniformly dispersed as single atoms within the carbon matrix, validating the single‐atom synthesis strategy via zeolite chemical substitution. Electron paramagnetic resonance (EPR) spectra at 77 K revealed a carbon radical signal at g = 1.996 across all samples (Figure 2g), indicating unpaired electrons and defect sites. The EPR intensity, correlating with unpaired electron concentration, was significantly lower in Fe‐N‐PCF than in N‐PCF and CF. This decrease is due to Zn volatilization from Fe─ZIF‐8 during high‐temperature calcination, leading to N‐vacancy defects. Fe atoms form strong covalent bonds with these defects, stabilizing single‐atom incorporation. The interaction between unpaired electrons at defect sites and Fe's d‐orbitals reduces the overall unpaired electron count, confirming successful Fe single‐atom incorporation. N vacancy defects in N‐PCF and CF are also validated by their EPR responses.

Furthermore, X‐ray photoelectron spectroscopy (XPS) was used to analyze the surface composition of the carbon fiber matrix in Fe‐N‐PCF.^[^ ^29^ ^]^ The spectrum confirmed the presence of C, N, and O heteroatoms, but no significant Fe peaks were detected due to its low concentration (Figures S9 and S10, Supporting Information).^[^ ^30^ ^]^ Inductively coupled plasma optical emission spectrometry (ICP‐OES) detected trace Fe (0.39 wt.%), consistent with XPS limits (<1 wt.%).^[^ ^24^ ^]^ Elemental content from XPS supported ICP‐OES findings (Table S2, Supporting Information). High‐resolution N 1s spectra showed five peaks: pyridinic N (≈398.2 eV), Fe‐N (≈399.4 eV), pyrrolic N (≈400.6 eV), graphitic N (≈401.8 eV), and oxidized N (≈403.4 eV), with pyrrolic N predominant (Figure 2h). Pyridinic and graphitic N anchor isolated Fe atoms and enhance conductivity through Fe‐N bonds. The Fe‐N bond at 399.4 eV indicates strong interactions between Fe and nearby N atoms, suggesting the formation of Fe‐N_x_ species, which enhances electrochemical performance. In Figure 2i, the C 1s spectrum shows peaks at 290.9, 287.9, 286.1, and 284.8 eV, corresponding to O─C≐O, C≐N, C─N, and C─C groups, confirming successful N incorporation into the carbon framework of Fe‐N‐PCF.^[^ ^31^ ^]^ Figure S11 (Supporting Information) presents Raman spectra of Fe‐N‐PCF, N‐PCF, and CF, showing the D band at 1350 cm⁻^1^ (defect‐induced disorder) and the G band at 1580 cm⁻^1^ (sp^2^‐hybridized carbon).^[^ ^32^, ^33^ ^]^ The D/G intensity ratios of 1.31, 1.29, and 1.07 reflect high structural disorder, which enhances K^+^ ion diffusion due to iron (Fe) and N doping. Moreover, Table S3 (Supporting Information) shows that the Fe‐N‐PCF exhibited slightly higher conductivity compared to the undoped N‐PCF. This small enhancement can be attributed to the incorporation of Fe as single atoms within the carbon framework. The design of single‐atom Fe and the existing N doping help to optimize the electronic structure, facilitating more efficient electron transport.

**Figure 3:**
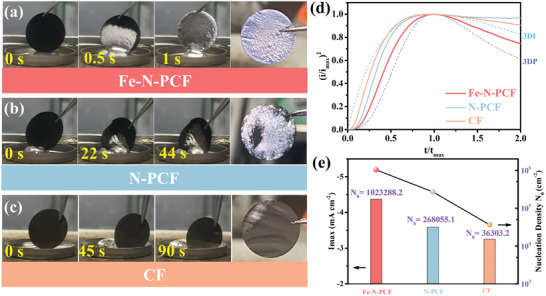
Images of K infusion into a) Fe‐N‐PCF, b) N‐PCF, and c) CF at different times. d) Dimensionless current‐time transient for the K deposition process in comparison with theoretical 3D models (I_max_: peak current; t_max_: time needed to achieve the peak current). e) Comparisons of maximum current of the potentiostat and nuclei number density calculated from CA curves.

Interconnected carbon fibers create capillary forces that improve molten K adsorption and infiltration, similar to lithium or sodium encapsulation. However, K's poor wettability on most carbon substrates is a challenge. To address this, structural optimization and chemical modification transformed CF into a K‐attractive, super‐wettable material, specifically as Fe‐N‐PCF. Infiltration experiments showed that Fe‐N‐PCF rapidly and uniformly adsorbed molten K at 150 °C,^[^ ^34^ ^]^ forming a smooth composite (Fe‐N‐PCF@K) in ≈1 s (**Figure** **3**a–c and Videos S1–S3, Supporting Information), significantly outperforming previous reports. The enhanced potassiophilicity of Fe‐N‐PCF is due to K‐attractive Fe‐N_4_ sites, which boost adsorption strength and diffusion rates. In addition, the hierarchical pore structure accelerates adsorption. In contrast, N‐PCF adsorbs K more slowly (≈6 s) and less uniformly. CF, with poor affinity, fails to fully adsorb molten K even after 90 s, underscoring the superior wettability of Fe‐N‐PCF.

Chronoamperometry (CA) was used to study the nucleation and growth of K on different substrates, critical for battery performance. A constant potential of −0.4 V (vs K^+^/K) was applied, and the resulting current transients (i‐t curves) were transformed into dimensionless plots (I/I_max_)^2^ (t/t_max_) to assess nucleation behavior. The current rose to a peak and then declined, indicating typical 3D nucleation and growth dynamics.

(5) $${\left(\frac{I}{I_{max}}\right)}^{2}=1.9542\frac{{\left[1-\exp\left(-1.2564\left(\frac{t}{t_{max}}\right)\right)\right]}^{2}}{\left(\frac{t}{t_{max}}\right)}$$

(6) $${\left(\frac{I}{I_{max}}\right)}^{2}=1.2254\frac{{\left[1-\exp\left(-2.3367{\left(\frac{t}{t_{max}}\right)}^{2}\right)\right]}^{2}}{\left(\frac{t}{t_{max}}\right)}$$

In the early deposition phase (t/t_max_ < 1), Fe‐N‐PCF exhibited progressive nucleation, with continuous nucleus formation and smooth, dense deposits. In contrast, N‐PCF and CF followed instantaneous nucleation, indicating rapid depletion of nucleation sites. In later stages (t/t_max_ > 1), both N‐PCF and CF maintained instantaneous nucleation, consistent with their deposition morphologies. Variations in nucleation and deposition overpotentials were observed, where lower nucleation overpotential shifted the mechanism from instantaneous to progressive. Notably, N‐PCF and CF deviated from theoretical curves, likely due to side reactions and dendrite formation. Nucleation densities from CA data show Fe‐N‐PCF has significantly higher nucleation density than N‐PCF and CF, with an order of magnitude increase (Figure 3e). This improvement contrasts with CF's K‐repellent nature, which results in loosely deposited dendrites. While N‐PCF improves nucleation density compared to CF, it still faces high energy barriers and uneven nucleation. In contrast, Fe‐N‐PCF, with Fe‐N_4_ single‐atom sites, reduces energy barriers for K nucleation and ion migration, promoting multidimensional growth. The hierarchical porous structure enhances mass transport, enabling dense, defect‐free, and uniformly distributed dendrite‐free K metal deposition.

**Figure 4:**
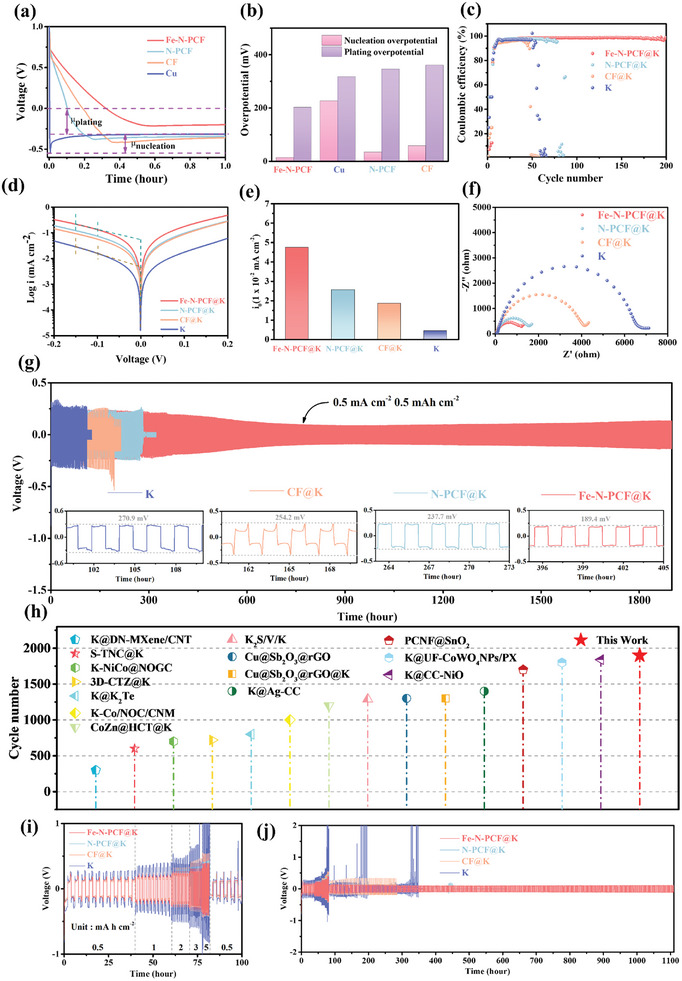
a) Voltage profiles of K nucleation on different hosts at a current density of 0.5 mA cm^−2^. b) Overpotentials of K deposition on different hosts. c) The corresponding CE performance of Fe‐N‐PCF, N‐PCF, CF, and bare K asymmetric cell at areal capacity of 0.5 mAh cm^−2^ with current density of 0.5 mA cm^−2^. d) Tafel plot obtain from LSV test of Fe‐N‐PCF@K, N‐PCF@K, CF@K, and bare K symmetric cell and e) the exchange current density of different hosts fitted from the Tafel plot. f) The EIS spectra of Fe‐N‐PCF@K, N‐PCF@K, CF@K, and bare K symmetric cell. g) Voltage profiles of symmetric cells with Fe‐N‐PCF@K, N‐PCF@K, CF@K, and bare K and corresponding enlarged profiles at current density of 0.5 mA cm^−2^ with a fixed areal capacity of 0.5 mAh cm^−2^. h) Comparison of cycle life time of symmetric cell with Fe‐N‐PCF and others advantage strategies to stabilize the PMB anode. i,j) Rate capability performance of symmetric cell at various current densities under a fixed areal capacity of 0.5 mAh cm^−2^.

To evaluate the nucleation and plating overpotentials of K metal on various substrates and assess the potassiophilicity of Fe‐N‐PCF, the initial nucleation overpotential (μ_nuc_) and deposition overpotential (μ_pla_) were measured in asymmetric cells at a constant current density of 0.5 mA cm⁻^2^ and an areal capacity of 0.5 mAh cm⁻^2^. The voltage‐time curves during potassium deposition revealed a sharp voltage drop followed by stabilization at a mass transfer‐controlled plateau (**Figure** **4**a), indicating nucleation and growth phases. The nucleation delay in carbon‐based materials, unlike copper foil, is attributed to SEI formation. The calculated nucleation overpotentials were 226 mV for copper foil, 57.3 mV for CNT, 33.5 mV for N‐PCF, and significantly lower at 13 mV for Fe‐N‐PCF, indicating reduced resistance for K nucleation (Figure 4b). In addition, μ_pla_ values showed that Fe‐N‐PCF (201.5 mV) had a lower mass transfer‐controlled potential compared to copper foil (317.7 mV), N‐PCF (345 mV), and CF (360.2 mV). The K^+^ diffusion coefficients (D^K+^) for CF, N‐PCF, and Fe‐N‐PCF were assessed using the galvanostatic intermittent titration technique (GITT) (Figures S12, Supporting Information). Fe‐N‐PCF demonstrated the highest D_K+_, ranging from 10^−7^ to 10^−8^, indicating superior diffusion kinetics, while N‐PCF exhibited restricted K^+^ diffusion due to the lack of Fe single‐atom site modification. CF had D_K+_ values between 10^−9^ and 10^−10^, leading to instantaneous nucleation for K deposition. These results confirm that Fe‐N‐PCF enhances charge transfer and K^+^ diffusion kinetics due to a higher density of active nucleation sites. While both N‐PCF and Fe‐N‐PCF feature hierarchical porous structures, the Fe‐N_4_ sites in Fe‐N‐PCF significantly boost K nucleation kinetics, highlighting the importance of single‐atom Fe doping. In contrast, CF's lower surface area and smooth structure hinder effective K deposition due to higher nucleation barriers.

Coulombic efficiency (CE) is critical for assessing the reversibility of PMAs during plating and stripping. Fe‐N‐PCF@K achieved an average CE of 98.63% at 0.5 mA cm^−2^ and 0.5 mAh cm^−2^, maintaining this high efficiency over 200 cycles (Figure 4c). In contrast, N‐PCF exhibited a CE of 98.24%, which declined after 75 cycles due to uncontrolled dendrite growth leading to uneven K deposition and “dead K.” The superior performance of Fe‐N‐PCF is attributed to Fe single atoms, which increase active site density, enhance ionic diffusion, reduce nucleation energy barriers, and ensure uniform K deposits, thus improving cycling stability. Bare K and CF showed lower CEs of 97.55% and 95.72%, respectively, with declines after 50 cycles due to low potassiphilic and insufficient active sites, promoting dendritic growth. To investigate ion diffusion kinetics, symmetric cells with various anodes were analyzed using linear sweep voltammetry (LSV). The curves indicated that Fe‐N‐PCF@K had the most symmetric profile and highest redox current, reflecting superior deposition/stripping kinetics (Figure S13, Supporting Information). Tafel plots revealed exchange current densities (j₀) of 4.7 × 10^−2^ mA cm^−2^ for Fe‐N‐PCF@K, outperforming N‐PCF@K (2.5 × 10^−2^ mA cm^−2^), CF@K (1.8 × 10^−2^ mA cm^−2^), and bare K (4.3 × 10^−3^ mA cm^−2^). These results highlight enhanced charge transfer dynamics at the Fe‐N‐PCF@K/electrolyte interface due to its hierarchical porous structure and Fe single‐atom sites (Figure 4d,e).^[^ ^35^ ^]^ Nyquist plots confirmed the lowest resistance for Fe‐N‐PCF@K (Figure 4f; Figure S14 and Table S4, Supporting Information).

In long‐term cycling tests, symmetric Fe‐N‐PCF@K cells demonstrated over 1900 h of stable operation at 0.5 mA cm^−2^ with a low overpotential of 189.4 mV (Figure 4g), outperforming previous studies (Figure 4h). The stable voltage distribution indicates effective dendrite suppression. Importantly, as shown in Figure S15 (Supporting Information), further analysis of the curves in Figure 4g reveals that the nucleation overpotential of Fe‐N‐PCF@K (0.309 V) is significantly lower than that of N‐PCF@K (0.528 V), indicating enhanced nucleation behavior. Furthermore, the incorporation of single‐atom Fe sites effectively optimizes the nucleation process. And, Fe‐N‐PCF@K exhibits lower deposition overpotential (0.268 V) compared to N‐PCF@K (0.527 V), exhibiting its superior kinetics in facilitating the deposition process. Initial cycles exhibited higher overpotentials due to side reactions, which decreased with cycling, likely due to improved SEI formation. The Fe‐N_4_ single‐atom sites significantly reduced nucleation overpotential and enhanced mass transfer, ensuring optimal active site exposure and improved kinetics. In contrast, N‐PCF@K, CF@K, and bare K had overpotentials of 237.7, 254.2, and 270.9 mV, respectively, with shorter cycle lives. Bare K showed significant voltage fluctuations within the first 20 h, indicating interface instability, a trend also observed in CF and N‐PCF after 171 and 210 h, respectively. This instability arises from uncontrolled plating/stripping and excessive dendrite growth. While N‐PCF enhances mass transfer, its limited nucleation sites and slower ion kinetics hinder battery lifespan. CF's lack of potassium‐philic sites and higher mass transfer resistance also led to earlier performance decay.

Rate performance tests at current densities from 0.5 to 5 mA cm^−2^ with a fixed capacity of 1 mAh cm^−2^ showed that Fe‐N‐PCF@K had exceptional rate capability and minimal polarization voltage hysteresis (Figure 4i). As current densities increased, voltage hysteresis rose to 188.1, 225.6, 272.1, 307.8, and 389.3 mV, respectively, demonstrating Fe‐N‐PCF's effectiveness in mitigating dendrite growth and regulating K deposition morphology. Even after returning to 0.5 mA cm⁻^2^, the symmetric Fe‐N‐PCF@K cell cycled for over 1100 h with stable voltage hysteresis (Figure 4j). In contrast, bare K, CF@K, and N‐PCF@K showed significant voltage drops at 347, 283, and 450 h, respectively, indicating short‐circuiting. Bare K exhibited large and unstable overpotential fluctuations at high current densities due to uneven deposition, while CF@K and N‐PCF@K had greater voltage hysteresis and unstable profiles. At 1 mA cm^−2^, Fe‐N‐PCF@K showed a stable voltage hysteresis of 220.4 mV and a cycling lifespan exceeding 550 h (Figure S16, Supporting Information), compared to just 150 h for bare K electrodes. Overall, Fe‐N‐PCF@K significantly outperformed other materials,^[^ ^35^, ^36^, ^37^, ^38^, ^39^, ^40^, ^41^, ^42^, ^43^, ^44^ ^]^ confirming that the combination of Fe single‐atom sites and a hierarchical porous structure enhances ion/charge kinetics, regulates K deposition, suppresses dendrite growth, and stabilizes the electrode interface for superior electrochemical performance.

**Figure 5:**
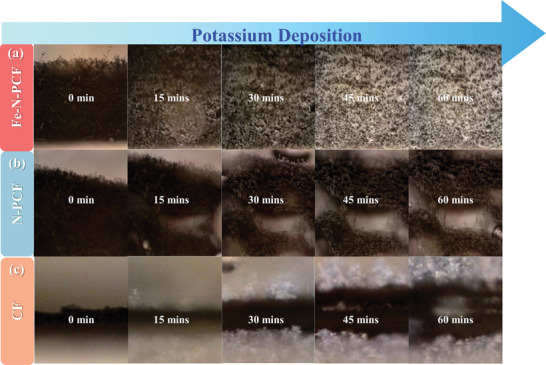
In situ OM observation of K deposition for 60 mins on a) Fe‐N‐PCF, b) N‐PCF, and c) CF.

To evaluate the nucleation and deposition behavior of K on Fe‐N‐PCF, in situ optical microscopy (OM) studies were performed (**Figure** **5**a–c; Figure S17, Supporting Information). Significant differences were observed between substrates. On CF, mossy dendrites appeared within 15 min, indicating rapid depletion of nucleation sites, consistent with the SH instantaneous nucleation model. Few K nuclei formed, suggesting CF quickly exhausted its nucleation sites. After 45 min, K continued growing on existing nuclei, forming needle‐like dendrites due to high energy barriers, resulting in uneven deposition, porosity, defects, and dead K. This compromised the SEI, increased side reactions, and degraded the electrode, raising the risk of short circuits. N‐PCF, despite its optimized porous structure, also faced high nucleation and diffusion energy barriers, limiting nucleation sites. After 15 min, uneven nuclei and K aggregation formed due to poor ion distribution. At 30 min, the emergence of new nuclei was overshadowed by dominant existing ones, leading to dendrite growth and impaired performance. By 60 min, non‐uniform K deposition resulted in dendrites, reducing electrochemical performance and cycle life, consistent with prior nucleation model analysis.

In contrast, Fe‐N‐PCF exhibited uniform K nucleation after 15 mins, with continual new nuclei formation at 30 min, indicative of progressive nucleation. Fe‐N‐PCF allowed for uniform deposition while preventing dendrite formation. The Fe‐N_4_ sites regulated K plating and stripping, while the hierarchical porous structure enhanced ion migration. After 60 min, the resulting K morphology was dense, smooth, and dendrite‐free, significantly improving electrochemical performance. In addition, K deposition morphology on copper foil indicated that mossy dendrites initiated formation after 15 mins (Figure S17, Supporting Information). At 30 mins of plating, both mossy and needle‐like dendrites expanded radially, increasingly covering the entire surface. This dendritic proliferation not only heightened the risk of short circuits but also compromised cycle stability. These findings further affirm that Fe‐N‐PCF effectively modulates K deposition behavior, stabilizes the electrode interface, and exhibits exceptional dendrite suppression capabilities, thereby enhancing the cycle life of PMA.

**Figure 6:**
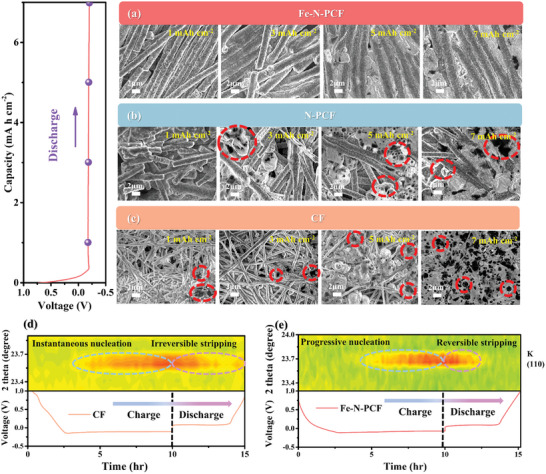
Ex situ SEM images of K metal plating on a) Fe‐N‐PCF, b) N‐PCF, and c) CF hosts at various deposition capacities from 1 to 7 mAh cm^−2^. Contour plot of Operando XRD pattern and corresponding voltage‐time profile of d) CF and e) Fe‐N‐PCF hosts.

In addition to in situ OM for K metal deposition observation, SEM provided at high‐magnification (**Figure** **6**a–c).^[^ ^45^ ^]^ Ex situ SEM images illustrate the morphology and distribution of K on CF, N‐PCF, and Fe‐N‐PCF at various deposition capacity (1, 3, 5, and 7 mAh cm^−2^). At 1 mAh cm^−2^, CF exhibits localized nucleation and agglomeration due to its K‐repellent nature, aligning with prior nucleation models. Some regions show loose, defective morphologies, likely from rapid dendritic growth. N‐PCF displays smoother deposition, reflecting enhanced mass transfer efficiency, though it still experiences some aggregation due to a high nucleation/diffusion energy barrier. Fe‐N‐PCF demonstrates a higher density of small K nuclei that diffuse laterally, forming a compact, uniform layer. At 3 mAh cm^−2^, CF reveals spherical protrusions consistent with dendritic formation, and N‐PCF shows significant aggregation and defects, indicating limited effectiveness of structural improvements alone in regulating deposition. In contrast, Fe‐N‐PCF maintains smooth and dense deposition, consistent with a progressive nucleation model. At a deposition capacity of 5 mAh cm^−2^, the CF substrate exhibited significant spherical dendrite formation, resulting in non‐uniform K deposition and compromising the stability of the SEI. This excessive dendrite growth raised safety concerns due to the risk of separator penetration. As the capacity increased to 8 mAh cm^−2^, CF displayed enhanced porosity and defects stemming from uneven deposition behaviors. Importantly, Fe‐N‐PCF maintained dense, smooth K coatings at both 5 and 8 mA h cm^−2^, effectively filling gaps between fibers and achieving a dendrite‐free surface. This performance underscores the efficacy of its hierarchical porous structure and abundant Fe‐N_4_ active sites in directing K deposition and enhancing electrochemical stability.

Operando XRD analysis was conducted to elucidate the electroplating and stripping behaviors (Figure 6d,e). At a current density of 0.1 mA cm^−2^, the XRD intensity contour plots and corresponding charge‐discharge curves revealed the crystalline evolution of K metal on both CF and Fe‐N‐PCF during the initial cycle. The crystalline evolution of K was tracked, revealing a new diffraction peak at 23.7°—indicative of the (110) plane of K—marking the onset of metal deposition. This peak appeared sooner in CF than in Fe‐N‐PCF, demonstrating the latter's superior potassiophilicity and availability of nucleation sites, which mitigated aggregation and promoted uniform deposition. Over time, the uniformly dispersed K nuclei expanded and merged laterally, minimizing K metal exposure during initial deposition. This process promoted the formation of amorphous K, resulting in a dense, uniform coating on the carbon substrate, consistent with progressive nucleation. During the stripping phase, the diffraction peak for Fe‐N‐PCF quickly vanished at a cut‐off voltage of 1 V, indicating no residual K and confirming excellent electroplating and stripping reversibility due to its structural features. In contrast, CF retained a persistent potassium signal during charging, indicating incomplete stripping and “dead” K formation. The potassiophobic nature of bare carbon substrates results in sparse nucleation sites and dendrite formation, hindering electroplating performance. N doping, such as pyridinic and graphitic N, improves potassiophilicity by increasing binding energy and adsorption, promoting nucleation, and reducing dendritic growth. However, N doping can compromise structural integrity and conductivity due to differences in electronegativity between K and N and changes in bond lengths. Incorporating single metal atoms to form MNx‐C sites offers a promising solution by optimizing K binding energy and stabilizing surface structures, improving deposition and extending battery performance and lifespan.

**Figure 7:**
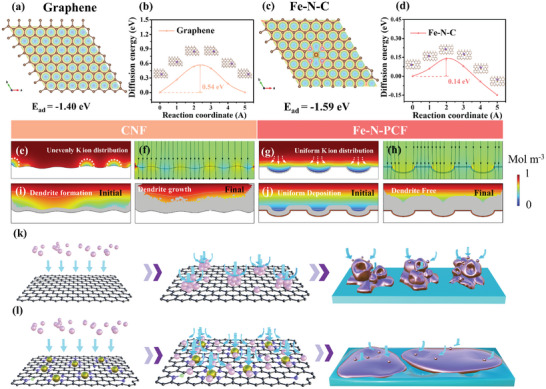
Electron density difference and adsorption energy (E_ad_) & K ion migration pathways and energy barriers on a,b) graphene and c,d) Fe‐N‐C. COMSOL Multiphysics simulation of K ion flux distribution at selected 2D phase‐field simulation states and current density distribution of on e,f) CF and g,h) Fe‐N‐PCF. The morphology evolution snapshots during K deposition on i) CF and j) Fe‐N‐PCF. Schematic illustration and mechanism of K atom diffusion modulated by pristine graphene k) and Fe‐N‐C sites distributed on graphene l), the possible plating/stripping mechanism on different K metal substrates of CF and Fe‐N‐PCF is also illustrated.

Density functional theory (DFT) was used to study interactions between K atoms and the Fe‐N‐PCF scaffold, revealing mechanisms of uniform K deposition and stripping (**Figure** **7**a–d). A single‐atom Fe‐N_4_ configuration was modeled alongside pristine graphene and pyridinic N‐doped carbon (Figure S18, Supporting Information). The adsorption models highlight the role of N coordination in enhancing the performance of carbon‐based materials in catalytic and electrochemical applications. DFT calculations show that K adsorption energy (E_ad_) on N‐doped graphene is significantly higher than on pristine graphene (−1.40 eV), indicating that N defects and Fe‐N_4_ sites favor improved K‐C interactions, facilitating K^+^ flux. Electronegativity differences among C (2.54), N (3.04), Fe (1.83), and K (0.82) influence K adsorption, with K's low electronegativity leading to strong binding at Fe‐N‐C sites. Pyridinic N shows the strongest N–K interaction, with the highest adsorption energy of −3.24 eV. Electrochemical performance tests demonstrate that Fe‐N‐PCF outperforms N‐PCF and CF in kinetic behavior and cycling stability, attributed to strong N‐K interactions that enhance structural stability. However, excessive bonding could risk structural collapse with increased metal deposition. Fe doping offers effective K nucleation sites while preserving structural integrity during deposition and stripping cycles. In contrast, weak K adsorption on graphene leads to poor deposition control and dendritic growth.

To evaluate the impact of surface structures on K adsorption, we calculated the electronic localization function (ELF) for graphene, pyridinic nitrogen, and Fe‐N_4_ (Figure 7a,c; Figure S19, Supporting Information). Without adsorbed K, strong Fe‐N interactions enable charge transfer to the graphene matrix. The Fe‐N_4_ configuration shows significant charge delocalization, optimizing K adsorption and nucleation. Charge density difference plots reveal electron transfer from K to graphene during adsorption, with substantial charge transfer in the Fe‐N_4_ configuration, confirming its effectiveness as an electron adsorption site (Figure S20, Supporting Information). Graphene's potassiophobic nature stems from its electron‐withdrawing C atoms, resulting in weaker K adsorption compared to Fe‐N‐PCF. Migration energy barriers play a crucial role in atomic diffusion, with lower barriers reducing surface tension and promoting uniform deposition. Figure 7b,d and Figure S21 (Supporting Information) show that K has the lowest migration barrier (0.14 eV) on Fe‐N_4_, while pyridinic nitrogen exhibits a higher barrier (1.42 eV), causing aggregation. Fe‐N bonds weaken potassium adsorption moderately but enhance lateral diffusion via effective charge transfer. Asymmetric battery tests show that Fe‐N‐PCF has significantly lower nucleation overpotential (13 mV) compared to N‐PCF (33.5 mV), indicating reduced energy barriers and improved charge transfer. The Fe‐N_4_ structure is crucial for uniform K deposition and enhanced cycling performance.

The current density distribution, K^+^ flux, and deposition morphology of CF, N‐PCF, and Fe‐N‐PCF were simulated using COMSOL Multiphysics (Figure 7; Figure S22–S26, Supporting Information). CF exhibited uneven K distribution and irregular current density due to surface roughness, leading to dendrite growth and unstable current (Figure 7e,f).^[^ ^46^ ^]^ The streamlines in the figures show how the electric field converges at protruding areas, causing ion accumulation and affecting deposition dynamics (Figure S22, Supporting Information). As K deposition advanced, the “tip effect” from uneven deposition worsened, distorting the electric field and ion flux, resulting in extensive dendrite growth and compromised electrochemical stability (Figure 7i). In contrast, Fe‐N‐PCF exhibited more uniform K^+^ distribution, with slightly higher current density within its porous structure (Figure 7h; Figure S23, Supporting Information). This uniformity, due to improved ion and electron transfer, supported effective K^+^ deposition both internally and externally. The Fe‐N coordination bonds regulated local current density and optimized electronic transfer, while the porous architecture increased surface area and reduced local current density, enabling dendrite‐free K deposition (Figure 7j). In N‐PCF (Figure S24, Supporting Information), the current density showed a significant gradient between pore edges and the interior, with higher current at curved edges, leading to K^+^ accumulation and dendrite formation (Figures S25 and S26, Supporting Information), limiting effective pore utilization. Moreover, we investigated the surface chemical composition of the cycled N‐PCF and Fe‐N‐PCF electrodes through XPS analysis (Figure S27, Supporting Information).^[^ ^47^ ^]^ The results reveal that Fe‐N‐PCF exhibits a significant reduction in the decomposition by‐products of organic solvents on its surface, suggesting Fe‐N_4_ might help suppress undesired side reactions.^[^ ^41^ ^]^ In contrast, N‐PCF shows a stronger K‐C peak, attributed to the continuous growth of the SEI layer caused by inactive K deposition.^[^ ^48^ ^]^ These differences in SEI composition highlight the beneficial role of Fe‐N_4_ sites in Fe‐N‐PCF, not only in promoting uniform K deposition but also in modulating the SEI chemistry, potentially improving the stability and reversibility of the system.

Figure 7k,l demonstrates that the Fe‐N_4_ single‐atom configuration, integrated with a porous architecture, forms potassiophilic sites that enhance ion flux, promoting uniform deposition and suppressing dendrite growth. In contrast, bare carbon in CF, with potassiophobic properties, hinders charge transfer and K adsorption due to a high nucleation energy barrier. This leads to uneven ion flux, uncontrolled aggregation, and a 3D instantaneous nucleation model (3DI),^[^ ^47^ ^]^ characterized by loosely packed dendrites that risk separator penetration, form dead K, and ultimately reduce cycle life through irreversible stripping behavior. The porous structure and Fe‐N_4_ sites enhance conductivity and electron transfer, promoting uniform nucleation with smaller K nuclei, consistent with heterogeneous nucleation theory. The low diffusion energy barrier of Fe‐N_4_ facilitates ion diffusion, enabling dendrite‐free growth and the formation of a dense plating layer (3DP model).^[^ ^47^ ^]^ This structure increases surface area, ensuring uniform current distribution and managing volumetric changes during cycling. The Fe‐N_4_ configuration lowers nucleation barriers, improves conductivity, and accelerates K^+^ diffusion, resulting in dendrite‐free K deposition.

**Figure 8:**
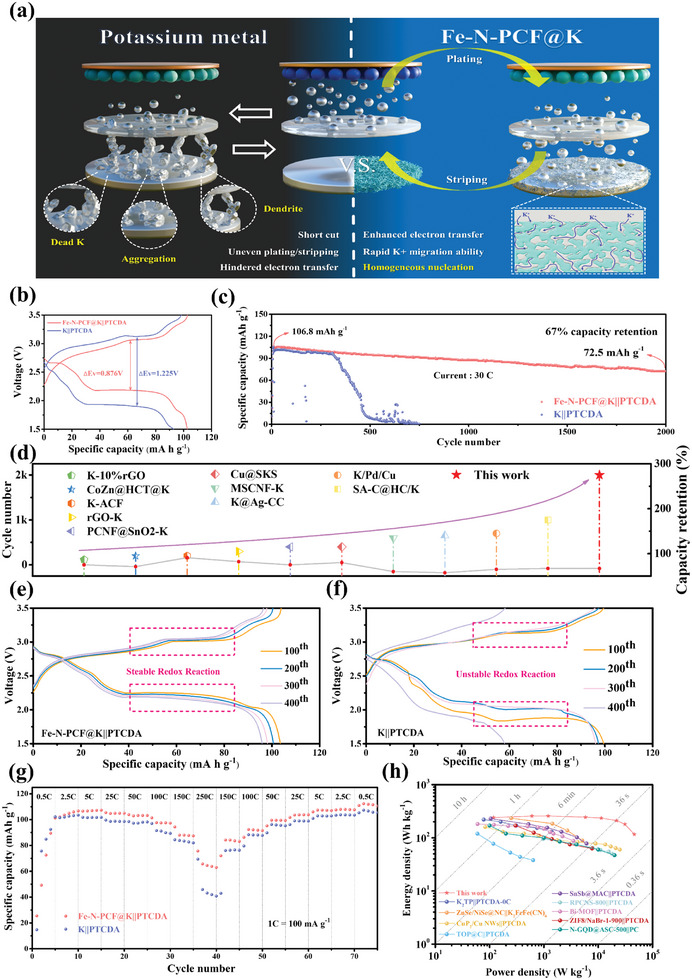
a) Schematic illustration of the working mechanism of the Fe‐N‐PCF@K electrode in PMB full cells. b) Voltage profile of bare K||PTCDA and Fe‐N‐PCF@K ||PTCDA full cell at 10th cycle. c) Long term cycling test of Fe‐N‐PCF@K||PTCDA and bare K||PTCDA full cells. d) Comparison of the recent literature on the cycle life and capacity retention of current K‐metal full cells. Charge and discharge voltage profile of e) Fe‐N‐PCF@||PTCDA and f) bare K||PTCDA full cells at 100th, 200th, 300th, and 400th cycles. g) Rate capability of bare K||PTCDA and Fe‐N‐PCF@K||PTCDA full cells. h) Ragone plots of Fe‐N‐PCF@||PTCDA full cells compared with other references.

Although Fe‐N‐PCF@K demonstrates impressive electrochemical performance in both half‐cell and symmetrical cell tests, its application as an anode in PMBs faces several key challenges (as shown in **Figure** **8**a). Using bare K as the anode, the lack of a structural framework during deposition causes substantial volumetric expansion, leading to unstable interfaces. This instability results in uneven deposition and stripping, promoting dendrite formation, agglomeration, and dead K, which degrade battery reversibility, increase failure risk, and severely shorten cycle life. Conversely, using Fe‐N‐PCF as the substrate enables stable and dense progressive nucleation of K metal. Fe‐N‐PCF@K not only supports reversible K^+^ storage but also stabilizes electrochemical reactions, accelerates redox kinetics, and maximizes K metal capacity utilization.

In the full battery assembly, Fe‐N‐PCF@K was used as the anode and PTCDA as the cathode, with bare K anodes as a control for comparative electrochemical analysis. As shown in Figure 8b, the 10th cycle GCD curves indicate that Fe‐N‐PCF@K exhibits significantly lower voltage polarization (1.225 V) compared to bare K (0.876 V), indicating improved redox kinetics. The degradation of the bare K||PTCDA full cell is due to inactive K accumulation, which hinders electron transport and depletes K resources. In long cycling tests (Figure 8c), the Fe‐N‐PCF@K||PTCDA full cell maintained a reversible capacity of 72 mAh g^−1^ after 2000 cycles at 30 C, with a capacity retention of 67%. This full cell demonstrated significantly better cycle longevity compared to recent PMB studies (Figure 8d).^[^ ^10^, ^44^, ^49^, ^50^, ^51^, ^52^, ^53^, ^54^, ^55^ ^]^ The improved cycling performance is due to stronger interactions between the Fe‐N‐PCF substrate and K, along with enhanced electron and ion transport. In contrast, the bare K||PTCDA full cell lasted only 500 cycles, with substantial capacity decay and just 9% retention after 500 cycles. The capacity decline was noted after 300 cycles, mainly due to K dendrite formation, causing inactive K buildup and capacity loss. These results confirm the effectiveness of the hierarchical porous structure and Fe‐N_4_ single‐atom configuration in lowering nucleation and diffusion energy barriers while improving electronic transfer. This design enables precise control of K nucleation, fostering a dendrite‐free, progressive mode that reduces performance degradation and safety risks in PMBs.

Figure 8e,f shows the GCD curves over various cycles. The Fe‐N‐PCF@K full cell benefits from favorable nucleation and deposition kinetics, leading to lower polarization voltage. After ten cycles, both full cells exhibit typical voltage profiles. However, by the 400th cycle, the bare K||PTCDA full cell shows a significant voltage drop, indicating unstable redox reactions and dendrite growth. In contrast, the Fe‐N‐PCF@K||PTCDA full cell maintains a stable voltage profile through the 200th, 300th, and 400th cycles. These results emphasized the ability of the hierarchical structure and Fe‐N_4_ single‐atom configuration to stabilize K metal kinetics, improving rate performance and cycle life. Figure 8g shows the Fe‐N‐PCF@K||PTCDA full cell achieving impressive reversible capacities at various current densities: 112, 108, 106, 104, 100, 93, 84, and 64 mA h g^−1^ (1 C = 100 mA g^−1^, based on cathode weight). While the bare K||PTCDA full cell delivers 107 mA h g^−1^ at lower rates, its performance declines sharply at 250 C due to dendrite growth, volumetric changes, and side reactions. The Ragone plot illustrates that the Fe‐N‐PCF@K||PTCDA full cell achieves a maximum energy density of 257 W h kg^−1^ and a power density of 46.2 kW kg^−1^ (based on cathode weight), outperforming previous studies on PTCDA (Figure 8g).^[^ ^32^, ^56^, ^57^, ^58^, ^59^, ^60^, ^61^, ^62^, ^63^ ^]^

## 3 Conclusion

This study elucidates the exceptional performance of PMBs using a hierarchical porous structure embedded with Fe‐N_4_ single‐atom sites. The material design combines a high electroactive surface area with flexibility, mechanical stability, and superior potassiophilicity, allowing for rapid molten potassium adsorption. These Fe‐N_4_ sites enhance K^+^ transport and electron conductivity, reducing nucleation and deposition overpotentials. Fe‐N‐PCF's low energy barriers for nucleation and diffusion significantly reduce overpotentials. Analysis using the SH model reveals an optimized nucleation mechanism, producing dense, uniform deposits without dendrites. The Fe‐N_4_ configuration also improves electrical conductivity and supports dendrite‐free growth. The Fe‐N_4_ sites serve as effective potassium‐active centers, lowering nucleation barriers, while the hierarchical porous structure enhances mass transport and active site exposure, improving kinetics and preventing dendrite formation. In situ OM and XRD confirm the excellent reversibility of Fe‐N‐PCF during plating/stripping, supporting stable cycling performance exceeding 1900 h. Testing of the Fe‐N‐PCF@K//PTCDA full cell shows stable capacity retention, faster redox kinetics, and reduced polarization, demonstrating its strong practical potential. To advance its practical applicability, future research should focus on long‐term stability testing under other electrochemical systems. Overall, Fe‐N‐PCF@K stands out as a promising anode material for PMBs, advancing high energy density and long‐lasting systems.

## 4 Experimental Section

### Materials

The chemicals used in this work such as methanol (99%), zinc nitrate hexahydrate (Zn(NO_3_)_2_·6H_2_O), Fe sulfate heptahydrate (FeSO_4_·7H_2_O), poly(vinylpyrrolidone) (PVP, Mw ≈ 55 000), 2‐methylimidazole, N, N‐dimethylformamide (DMF, 99.5%), polyacrylonitrile (PAN, MW = 150 000), perylene‐3,4,9,10‐tetracarboxylic dianhydride (PTCDA, 97%), sodium carboxymethyl cellulose (NaCMC, Mw ≈ 700 000), diethyl carbonate (anhydrous, ≥99%) and potassium (chunks, in mineral oil, 98% trace metals basis) were purchased from Sigma–Aldrich and used as received without any further purification. Potassium bis(fluorosulfonyl)imide (KFSI, 97%) was purchased from Combi‐Blocks. Super‐P, ethylene carbonate (EC) and CR‐2032 coin‐type cell were purchased from shining energy. Copper foil and aluminum foil were purchased from Chang Chun Group. Glass fiber was purchased from Advantec. Deionized water (Millipore Milli‐Q, resistivity ≥ 18.2 MΩ cm^−1^) was use in all our experiments.

### Preparation of CF

CF were fabricated using electrospinning process and followed by high‐temperature carbonization. For electrospinning, the precursor solution was prepared by dissolving PAN into DMF and mechanically stirred at 50 °C overnight. Subsequently, electrospinning process was carried out by applying a voltage of 16 kV and a distance of 17 cm between the needle and the grounded roller. The flow rate of syringe pump was set to be 1.5 mL h^−1^. The electrospun PAN mat was first stabilized at 280 °C for 3 h in air with a heating rate of 2 °C min^−1^ followed by high‐temperature carbonization at 800 °C for 2 h with a heating rate of 5 °C min^−1^ under nitrogen atmosphere.

### Preparation of Fe‐N‐PCF and N‐PCF

First, the obtained Fe─ZIF8 nanoparticles (0.45 g) were homogeneously dispersed in 3.6 mL DMF. Subsequently, 0.36 g of PAN was added to the above solution and stirred for 24 h at 50 °C. The homogeneous solution was filled into a 10 mL plastic syringe with a 21‐gauge nozzle and then electrospun. The flow rate, voltage, and distance between the collector and nozzle were set to 1.5 mL h^−1^, 16 kV, and 17 cm, respectively. The obtained nanofibers were first subjected to pre‐oxidation for 2 h at 150 °C (2 °C min^−1^) in air and then held at 800 °C (2 °C min^−1^) for 3 h in an Ar atmosphere to carbonize the PAN, producing Fe‐N‐PCF, the synthesis process of N‐PCF follows the above steps but substitutes the addition of Fe─ZIF8 with ZIF8 instead.

### Preparation of Fe‐N‐PCF@K, N‐PCF@K, CF@K Composite Anode

Molten K was prepared by placing K metal in a stainless‐steel base on a hot plate with a temperature of over 100 °C. Then, a piece of Fe‐N‐PCF, N‐PCF, CF mat came contact with the molten K to form Fe‐N‐PCF@K, N‐PCF@K, CF@K composite. The preparation process was conducted in an argon‐filled glove box (H_2_O, O_2_< 0.1 ppm).

### Material Characterization

All materials were characterized by using SEM (HITACHI‐SU8010), TEM (JEOL, JEM‐ ARM200FTH), and XRD (Bruker D8 ADVANCE). To obtain HRSEM images of materials, a HITACHI‐SU8010 field‐emission SEM with 10 kV accelerating voltage and 8 mm working distance were utilized. Meanwhile, XRD patterns were obtained by a Bruker D8 ADVANCE diffractometer equipped with Cu Kα radiation. The K‐edge XAS of Fe‐N‐PCF were measured in fluorescent mode at the beamline of TPS 44A at the National Synchrotron Radiation Research Center (NSRRC) in Taiwan. All the XAS data were analyzed by Athena software (version 0.9.26). The unpaired electrons in the e_g_ orbital of Fe atom in Fe‐N‐PCF was characterized by EPR spectrometer (Bruker, ELEXSYS E‐580, NTHU). Fe‐N‐PCF were characterized by high‐resolution XPS (ULVAC‐PH, PHI QuanteraII, NTHU). The Confocal Raman Microscope was used for pristine materials (PTT, MRID). Metal content in catalysts was determined by ICP‐OES (Agilent 725, NTHU).

### Electrochemical Characterization

All the electrochemical performance test was conducted with the CR2032 coin cells. The coin cells were assembled in the Ar‐filled glovebox (O_2_ < 0.1 ppm, H_2_O < 0.1 ppm, M. Braun UNILAB), 1 M KFSI in EC/DEC (1: 1, vol%) was selected as electrolyte and glass fiber as separator. The dosage of electrolyte was 180 µL for each coin cells assembly. The symmetric cells were assembled by two identical electrodes (Fe‐N‐PCF@K, N‐PCF@K, CF@K). The asymmetric cells were assembled with the copper foil as current collector and different K metal host (Fe‐N‐PCF@K, N‐PCF@K, CF@K, bare K). To stabilize the SEI layer, all the asymmetric cells were activated by cycling with the working window of 0.01–1 V with current density 0.1 mA cm^⁻2^ before K plating. The cut‐off voltage of K stripping on different hosts were set to 1 V. CV and EIS curves were obtained from the VMP3 workstation (Bio‐Logic‐Science) with a potential window of 0.01–3.00 V and a frequency of 600 kHz–50 mHz. Galvanostatic discharge/charge measurements were performed on Neware CT‐4000 galvanostat. The Operando OM observation was conducted with the customized holder (BJSCISTAR, LIB‐MS‐II) at the current density of 1 mA cm^−2^. The Operando XRD was investigated by Bruker D8 ADVANCE diffractometer. Different host (Fe‐N‐PCF@K, N‐PCF@K, and CF@K) were used as working electrode, K foil as counter electrode and the same electrolyte as asymmetric cell without activation before testing.

## Conflict of Interest

The authors declare no conflict of interest.

## Author Contributions

T.‐C.L. and Y.‐C.Y. equally contributed to this work. T.‐C.L., Y.‐C.Y., and H.‐Y.T. came up with the original idea and designed the experiments. T.‐C.L. performed the methodology, investigation and data curation, conducted experiments, and wrote the manuscript. Y.‐C.Y. performed the investigation and data curation, wrote the manuscript, and visualized the idea for the study. H.‐Y.T. contributed to writing—review & editing, handled funding acquisition, and provided resources. All authors reviewed and approved the final draft.

## Supporting information



Supporting Information

Supporting Information

Supporting Information

Supporting Information

## Data Availability

The data that support the findings of this study are available from the corresponding author upon reasonable request.
