# Supplementary material for: Optimized K+ Deposition Dynamics via Potassiphilic Porous Interconnected Mediators Coordinated by Single‐Atom Iron for Dendrite‐Free Potassium Metal Batteries
Source: Adv Sci (Weinh). 2025 Jan 9;12(8):2413804. doi: 10.1002/advs.202413804 (PMC11848633; doi:10.1002/advs.202413804)
Supplement: Supplementary file 1 — Supporting Information [file ADVS-12-2413804-s002.docx]

**Supporting Information**

Optimized K^+^ Deposition Dynamics via Potassiphilic Porous Interconnected Mediators Coordinated by Single Atom Iron for Dendrite-Free Potassium Metal Batteries

*Tzu-Chi Lin, Yi-Chun Yang, and Hsing-Yu Tuan **

T.-C. Lin, Y.-C. Yang, H.-Y. Tuan

Department of Chemical Engineering, National Tsing Hua University, Hsinchu 30013, Taiwan

E-mail: hytuan@che.nthu.edu.tw

**Experimental section**

**Synthesis of Fe-ZIF-8 & ZIF-8**

Typically, Zn(NO_3_)_2_·6H_2_O (0.575 g) and FeSO_4_·7H_2_O (0.019 g) were dissolved into 50 mL of methanol to form a solution. 50 mL of methanol containing 2-methylimidazole (0.66 g) and PVP (0.6 g) were poured into Zn(NO_3_)_2_ and FeSO_4_ solution. The mixture was kept stirring at room temperature for 24 h. The resulting white precipitates were collected by centrifugation, washed with methanol three times, and finally dried at 80 °C overnight. The products were named as Fe-ZIF-8. The synthesis process of ZIF-8 follows the above steps but omits the addition of FeSO_4_·7H_2_O.

**Computational Methods**

**First principal calculation**

We used the DFT as implemented in the Vienna Ab initio simulation package (VASP) in all calculations. The exchange-correlation potential is described by using the generalized gradient approximation of Perdew-Burke-Ernzerhof (GGA-PBE). The projector augmented-wave (PAW) method is employed to treat interactions between ion cores and valence electrons. The plane-wave cutoff energy was fixed to 400 eV. Given structural models were relaxed until the Hellmann-Feynman forces smaller than -0.02 eV/Å and the change in energy smaller than 10^-5^ eV was attained. The Γ-centered 3*3*1 k-point grids were applied for the Brillouin zone sampling for the geometry optimization. The long-range van der Waals interaction is described by the DFT-D3 approach. The diffusion barrier of a potassium atom on the three surfaces was calculated by climbing-image nudged elastic band (CI-NEB) method.

The adsorption energy (E_ads_) was calculated as:

E_ads_ = E_(system)_-E_(catalyst)_ -E_(species)_

where E_(system)_, E_(catalyst)_, and E_(species)_ are the total energy of the optimized system with adsorbed species, the isolated catalyst, and species, respectively.

**COMSOL simulation**

The simulation was conducted using the Tertiary Current Distribution physics interface within the Electrochemistry Module of COMSOL Multiphysics software to simulate the surfaces of three different electrodes. This model is based on the “Tertiary Current Distribution” interface to calculate the current in both the electrolyte and the electrodes. Thus, the electrolyte current is solved according to Ohm's law. One electrode is grounded, while the other is set to the cell potential to satisfy the total current condition.

The electric field is governed by the continuity equation of current density:

$$i= -F\sum-Z_{i}^{2}m_{i}Fc_{i}\nabla\emptyset_{l}$$

i represents the current density vector (SI unit: A/m²); zᵢ is the ion charge number; mᵢ denotes the mobility (SI unit: mol·m²·s·V⁻¹·A⁻¹); F is the Faraday constant (SI unit: A·s/mol); ϕₗ is the ionic potential, and cᵢ is the ion concentration. The conservation of current density is also satisfied.

$$\nabla\cdot i=0$$

In the simulation, the electrode kinetics occurring at the electrode surface embedded in the electrolyte is described using the Butler-Volmer formalism, with the exchange current density of the oxidation reaction considered concentration-dependent. The current density at the electrode surface is referenced by the Butler-Volmer equation (you can look up the definitions of related variables online):

$$i_{a}= i_{0}(\frac{c}{c_{0}}exp(\frac{\eta\left( 1-\beta\right)F}{RT})-exp(\frac{\eta\beta F}{RT})$$

The initial value of the electrolyte potential is set to be equivalent to the open-circuit potential of the battery (i.e., when the potential is unactivated). The following is the definition of overpotential:

η = φ*_s_* - φ*_l_* - *E*_eq_

Where η is the overpotential, φ*_s_* is the electrode potential, φ*_l_* is the electrolyte potential, and *E*_eq_ is the equilibrium potential. The transport of dissolved ions generated by the electrode reaction in the electrolyte is modeled through transient simulations of the "dilute species transfer" interface, which assumes that ion transport can be described by diffusion according to Fick's laws. Additionally, the mass transfer caused by both diffusion and migration is considered.

$$-D\nabla C-zmFc\nabla\phi_{l}=\mathbf{N}$$

c represents the ion concentration (SI unit: mol/m³), z is the valence, D is the diffusion coefficient (SI unit: m²/s), m is the mobility (SI unit: mol·m²/(s·V·A)), F is Faraday's constant (SI unit: A·s/mol), and $\phi_{l}$ is the ion potential.

In terms of boundary conditions, we set the reference potential at the bottom to be zero, while the top boundary is set to an average current density of 2.50 mA/cm². Additionally, the conductivity of the electrode material is set to 1e^7^ S/m, the conductivity of the electrolyte is set to 4.5 S/m, and the diffusion coefficient of the ions is set to 1e^-9^ m²/s.


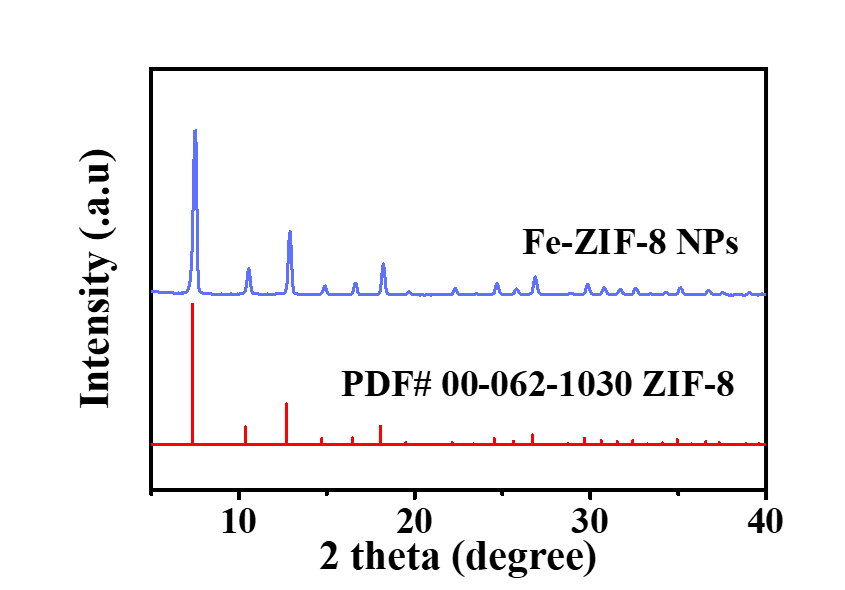


**Figure S1.** XRD pattern of Fe-ZIF-8 NPs.


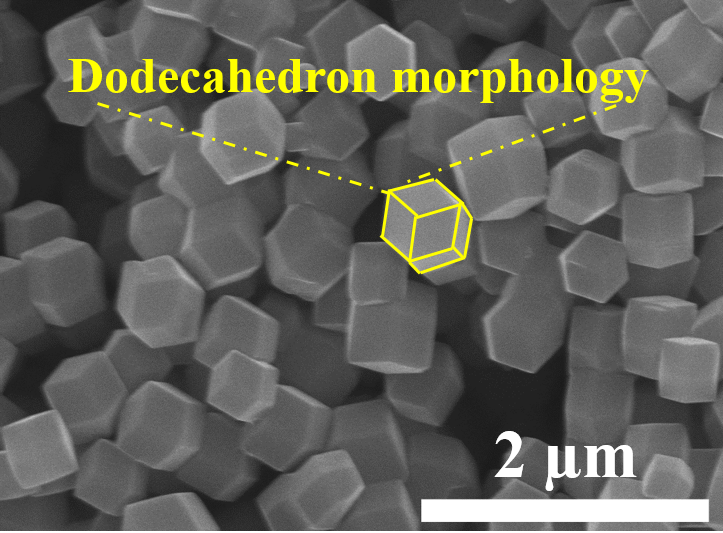


**Figure S2.** SEM images of Fe-ZIF-8.


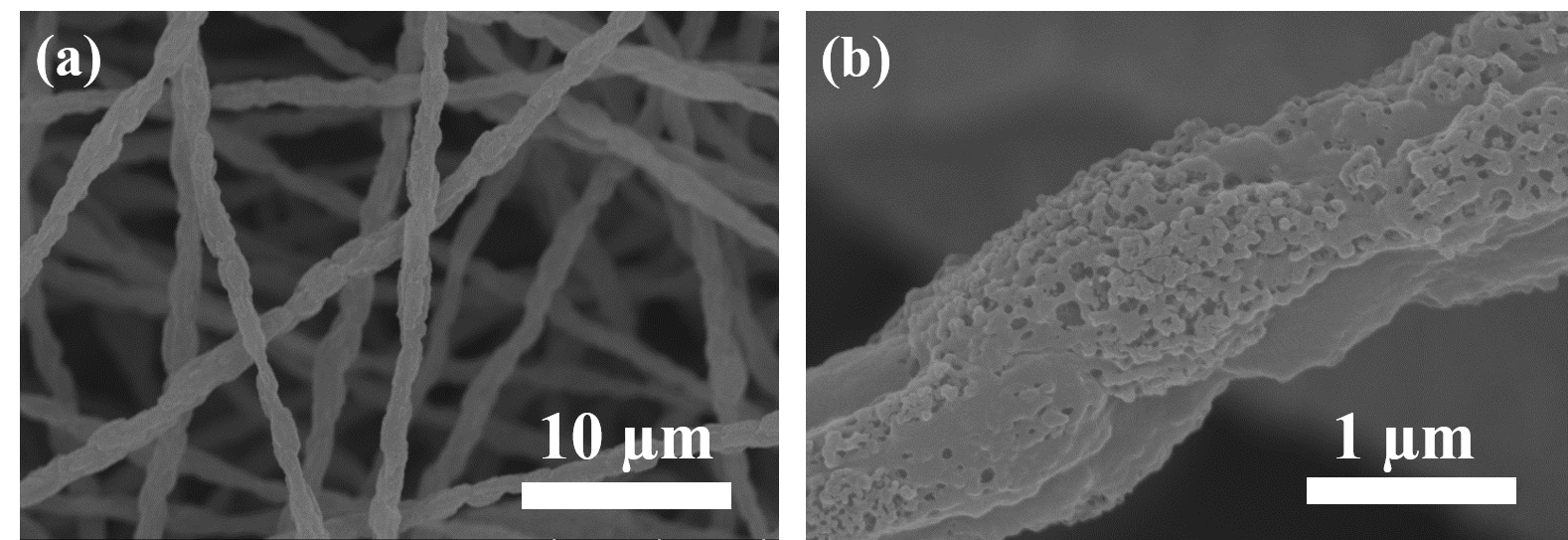


**Figure S3.** (a,b) SEM images of N-PCF.


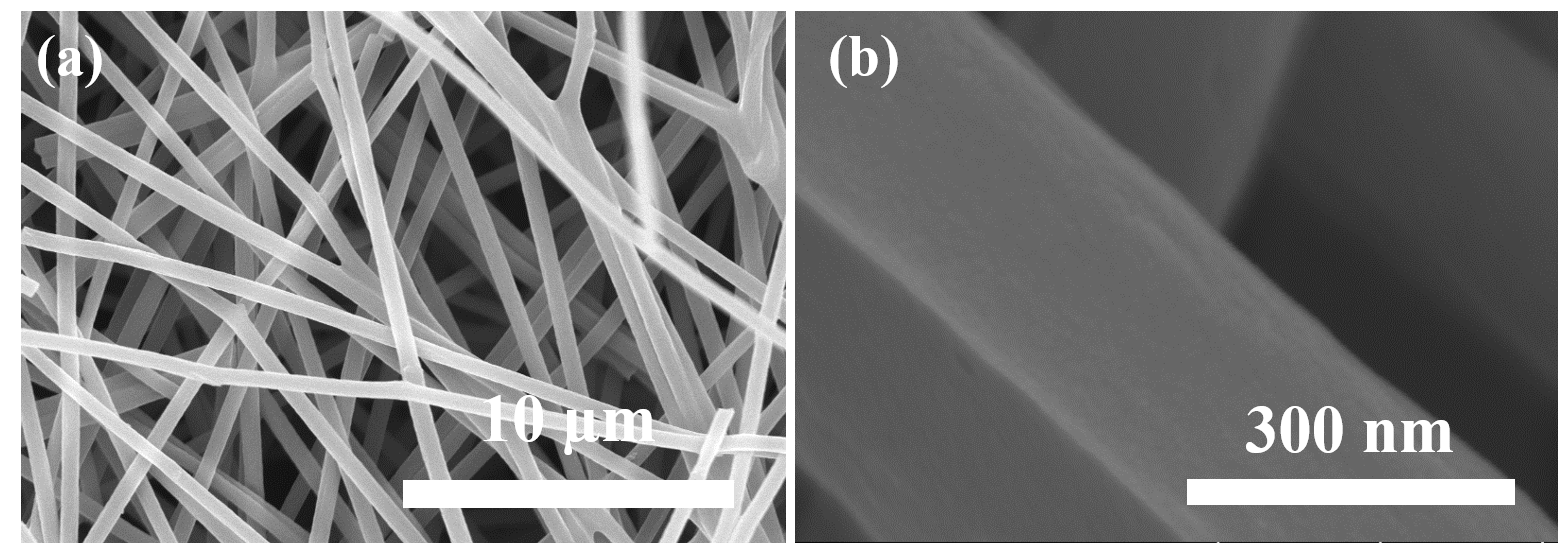


**Figure S4.** (a,b) SEM images of CF.


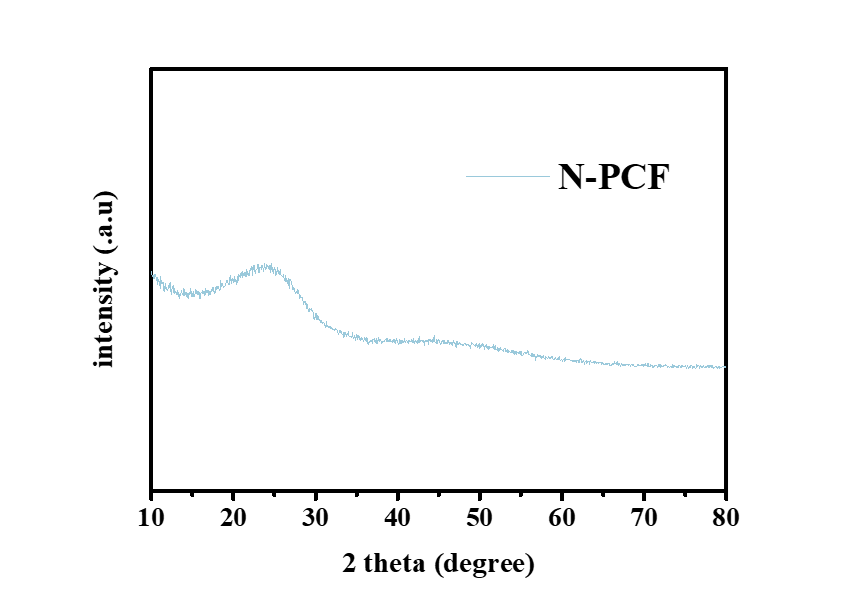


**Figure S5.** XRD pattern of N-PCF.


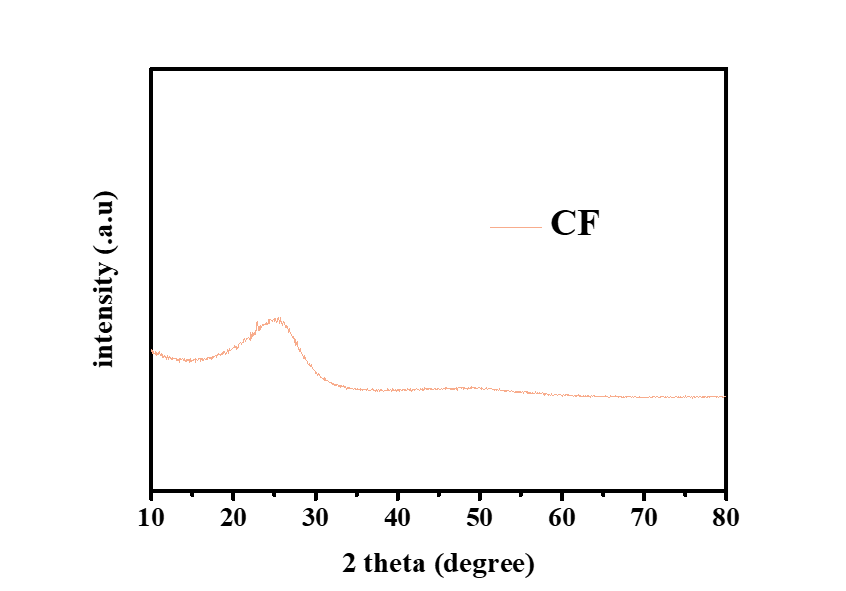


**Figure S6.** XRD pattern of CF**.**


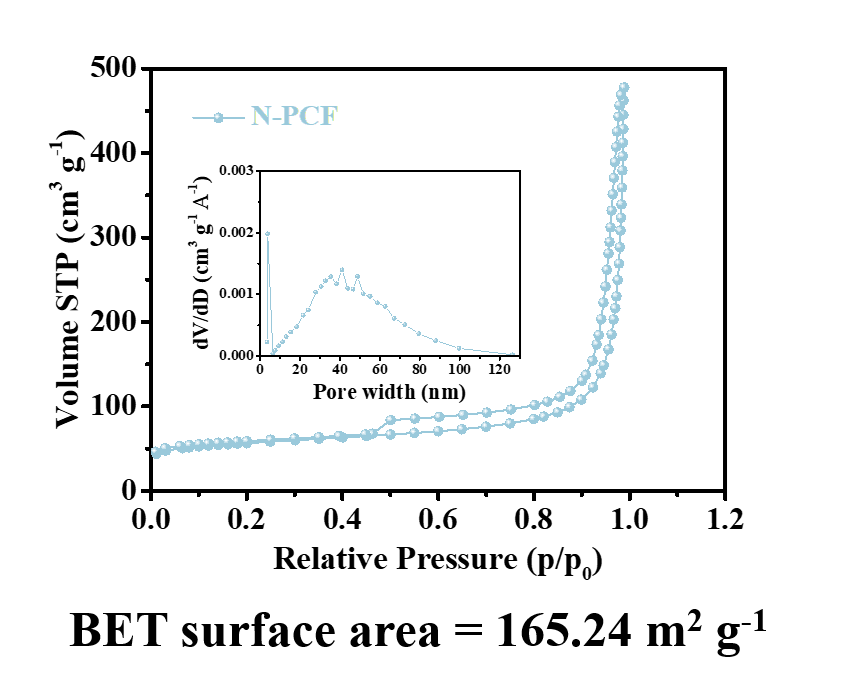


**Figure S7.** Nitrogen adsorption-desorption isotherms curve and Pore size distribution of N-PCF.


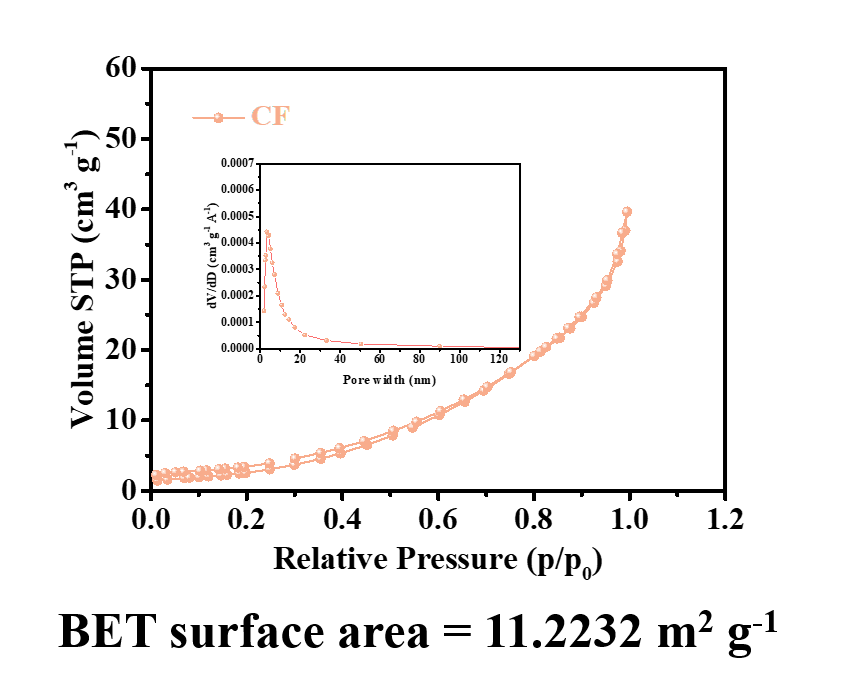


**Figure S8.** Nitrogen adsorption-desorption isotherms curve and Pore size distribution of CF.


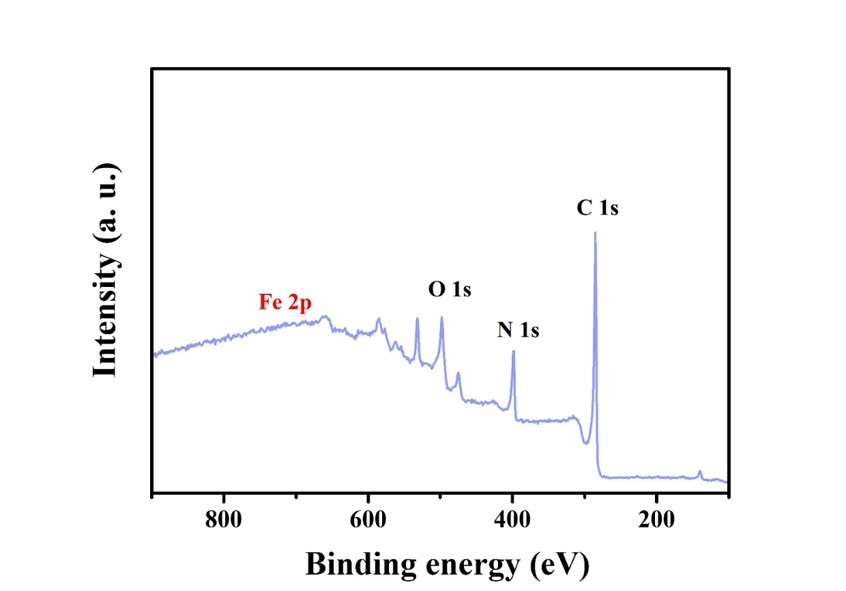


**Figure S9.** XPS survey spectrum of Fe-N-PCF.


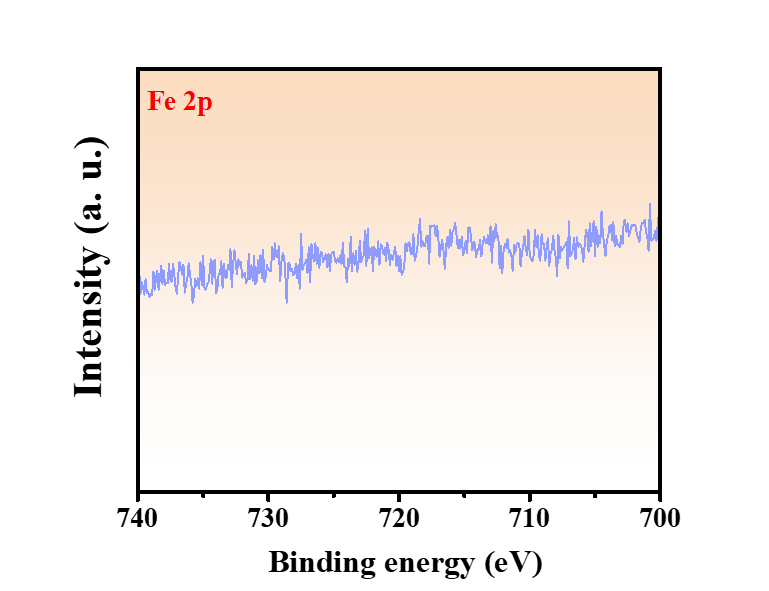


**Figure S10.** Fe 2p XPS spectrum of Fe-N-PCF.

**
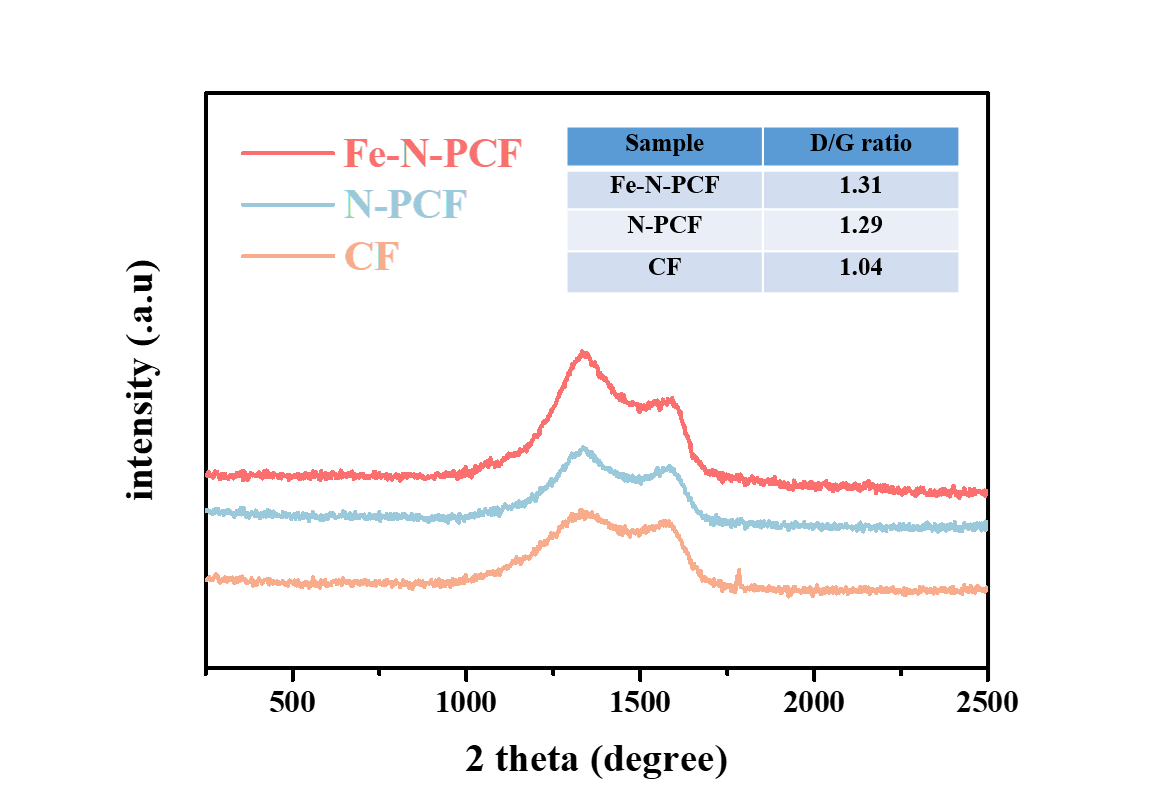
Figure S11.** Raman spectra of CF, N-PCF and Fe-N-PCF.


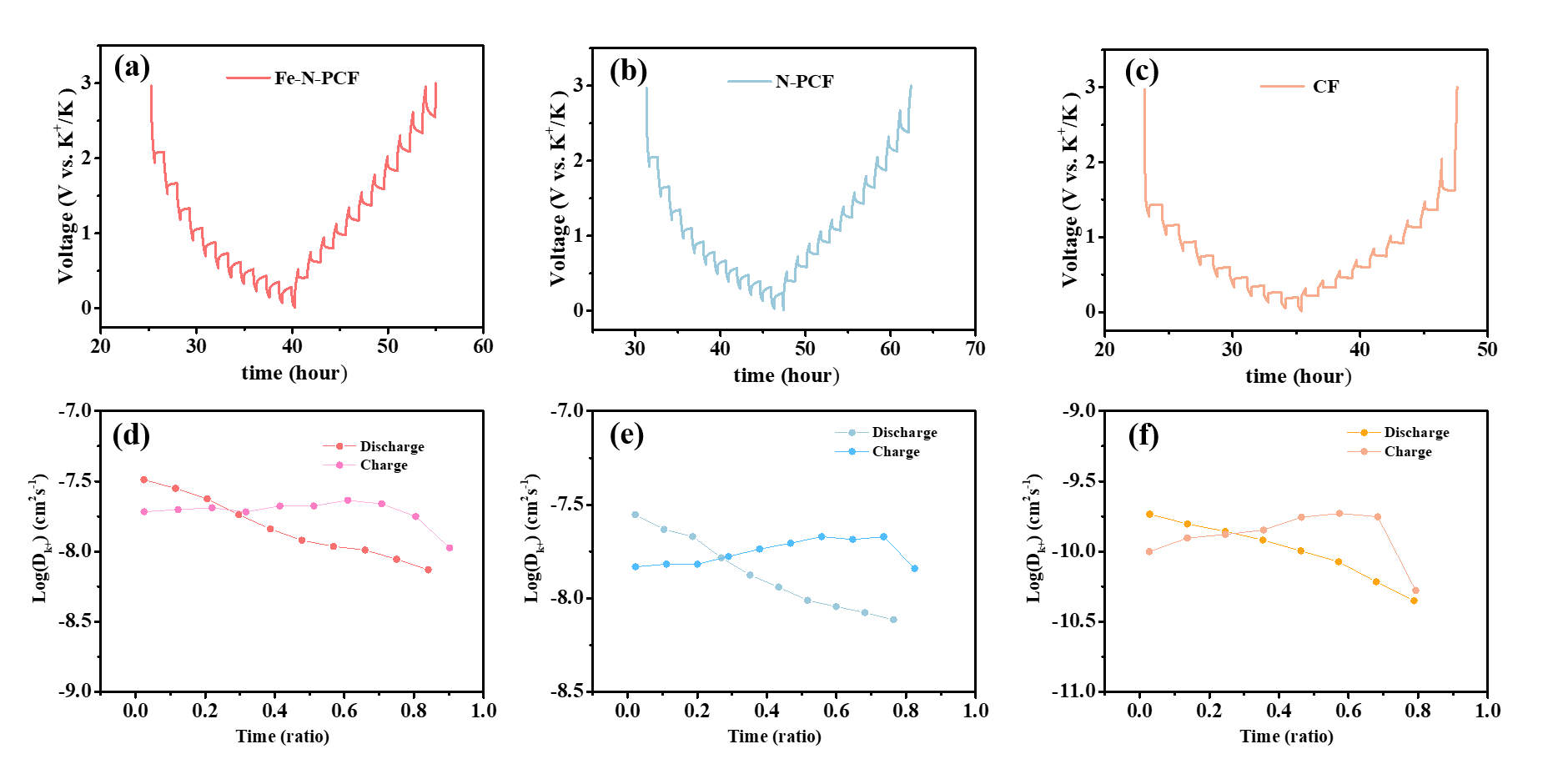


**Figure S12.** GITT profile of (a) Fe-N-PCF, (b) N-PCF and (c) CF substrate. Corresponding diffusion coefficient of (d) Fe-N-PCF, (e) N-PCF and (f) CF substrate.


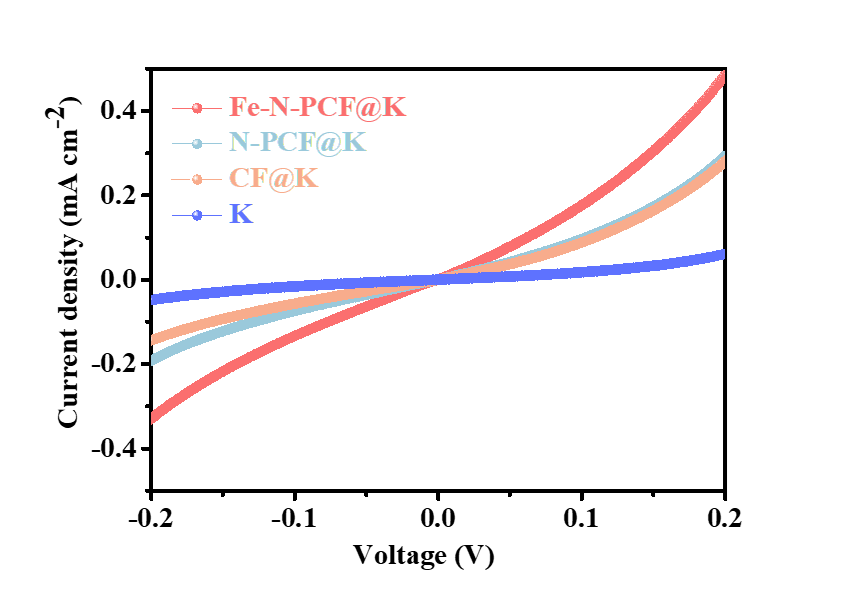


**Figure S13.** Linear sweep voltammetry (LSV) of Fe-N-PCF、N-PCF、CF and bare K symmetric cell at a scan rate of 1 mV s^−1^.


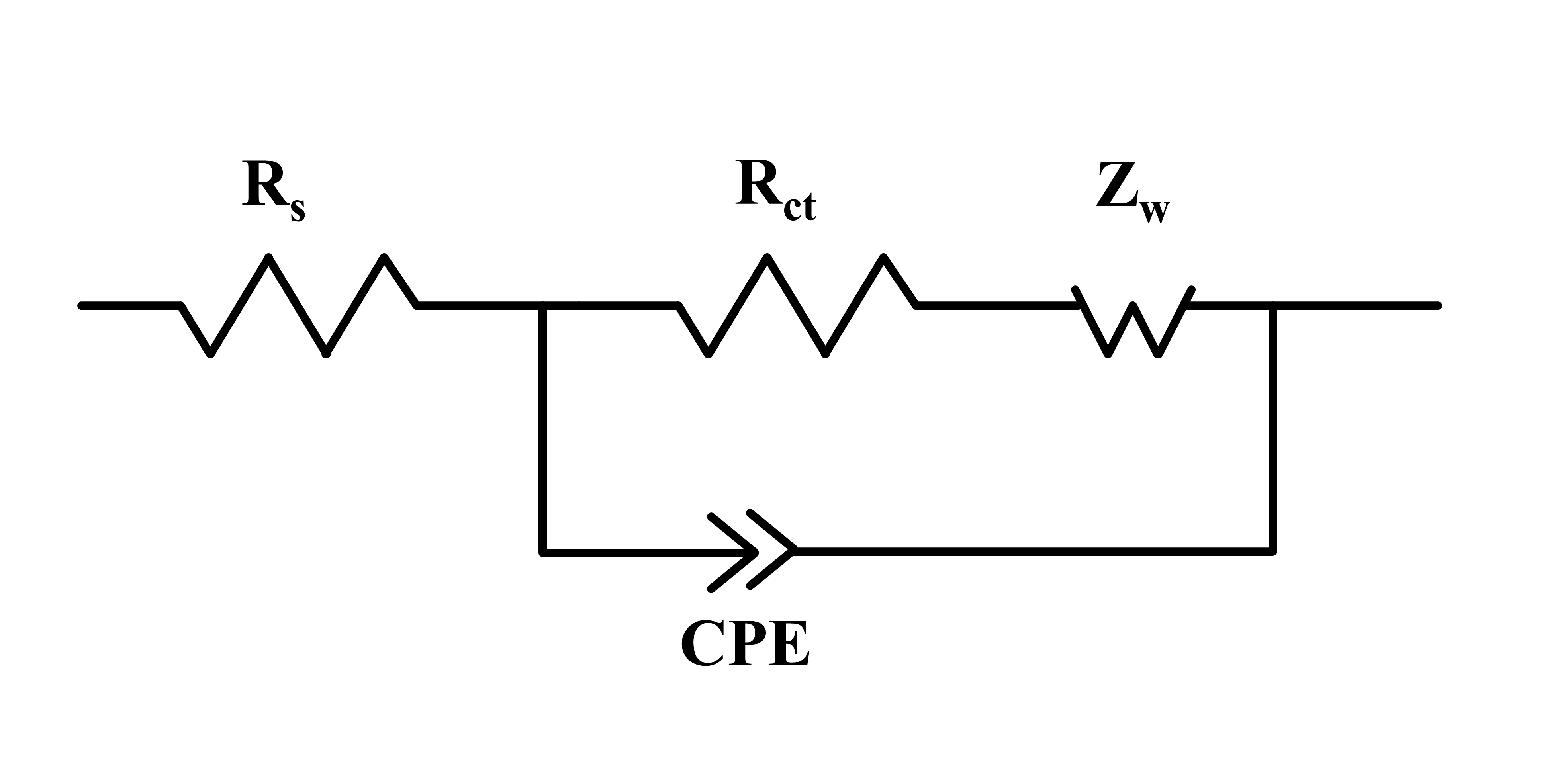


**Figure S14.** The equivalent circuit model.

**

**

**Figure S15.** Voltage profiles of K nucleation on N-PCF@K and Fe-N-PCF@K at a current density of 0.5 mA cm^−2^.


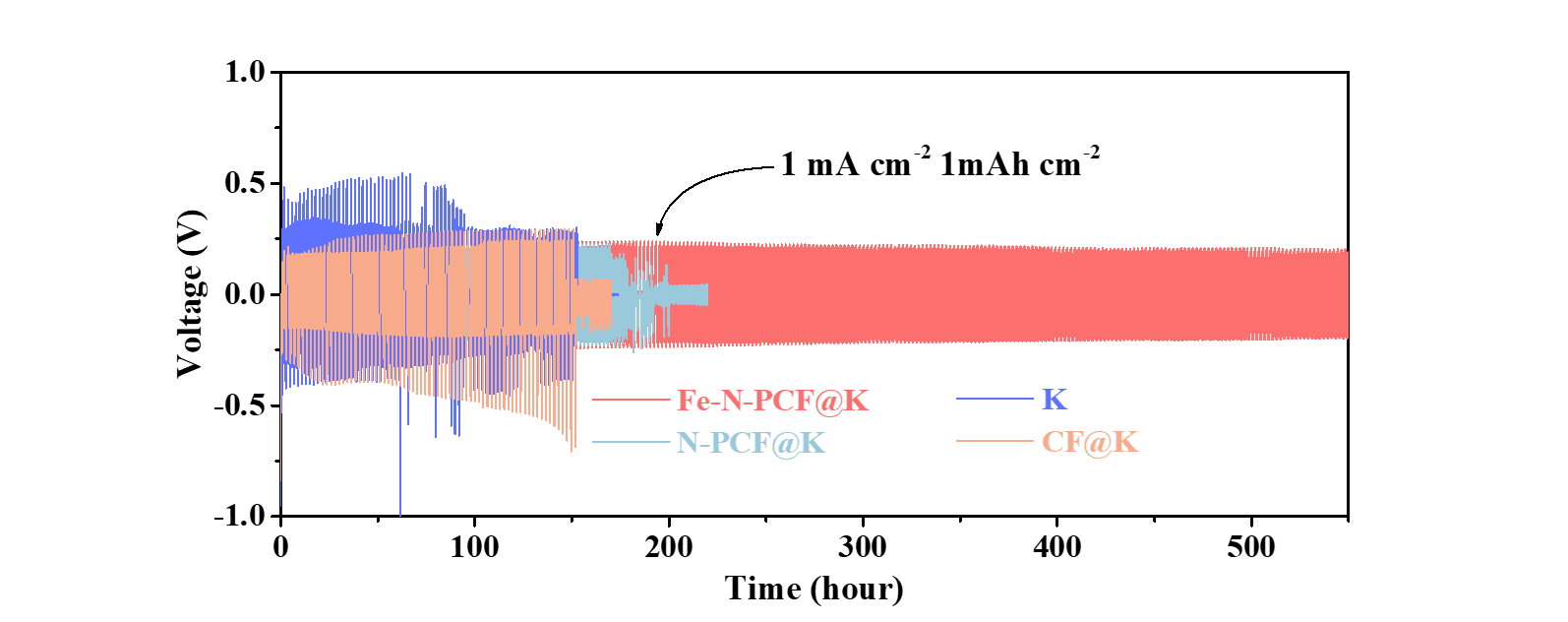


**Figure S16.** Voltage profiles of symmetric cells with Fe-N-PCF@K、N-PCF@K、CF@K and bare K and corresponding enlarged profiles at current density of 1 mA cm^−2^ with a fixed areal capacity of 1 mAh cm^−2^.


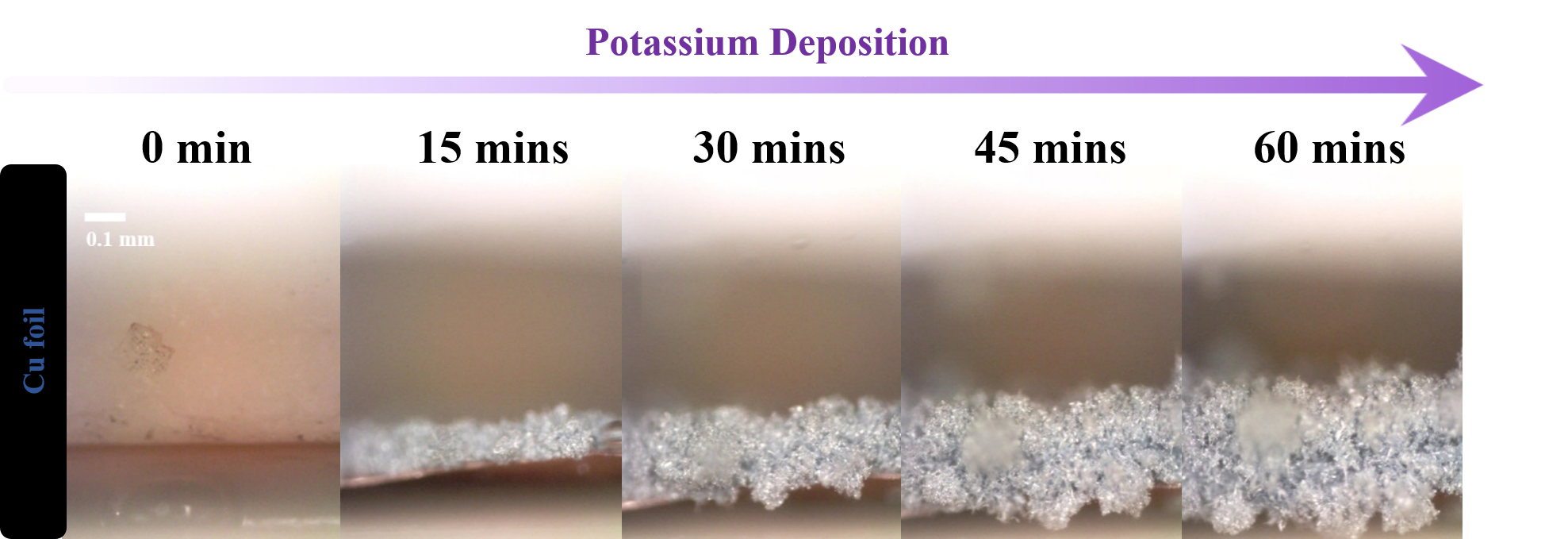


**Figure S17.** Operando optical microscopy observation of K plating on Cu foil


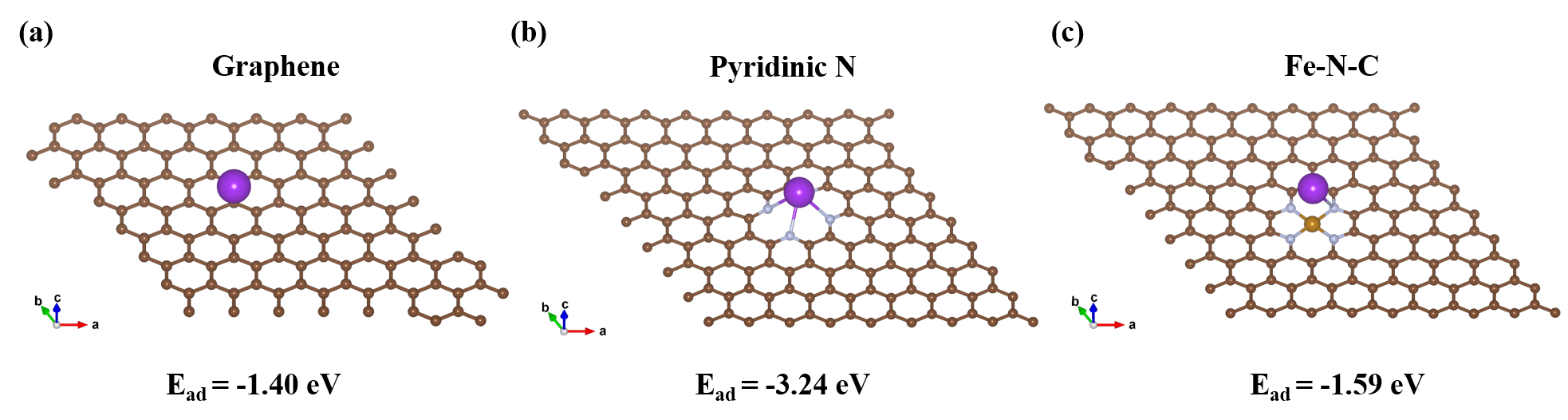


**Figure S18.** The optimized adsorbed configurations and adsorption energy of non-metallic atom binding carbon with K atom. a) Graphene, b) Pyridinic N, c) Fe-N-C


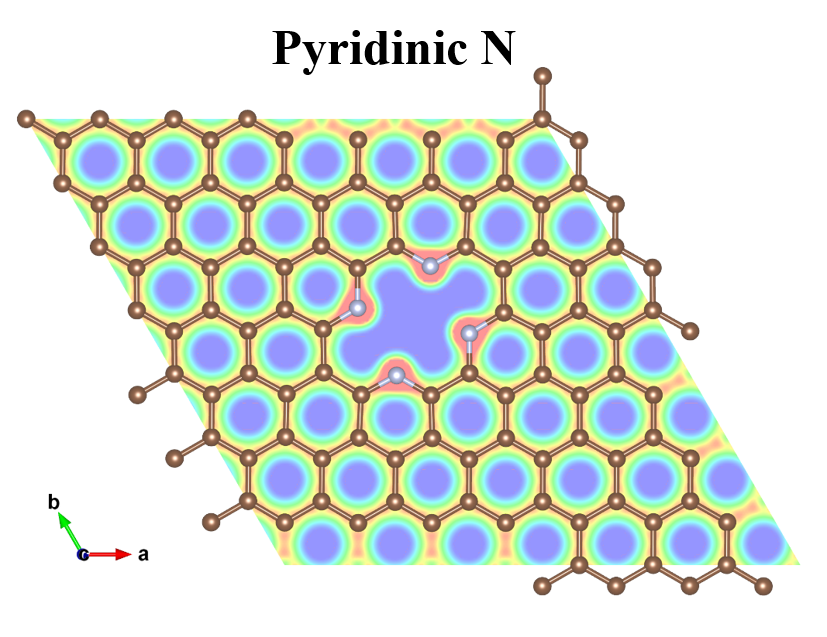


**Figure S19.** Electron density difference of Pyridinic N.


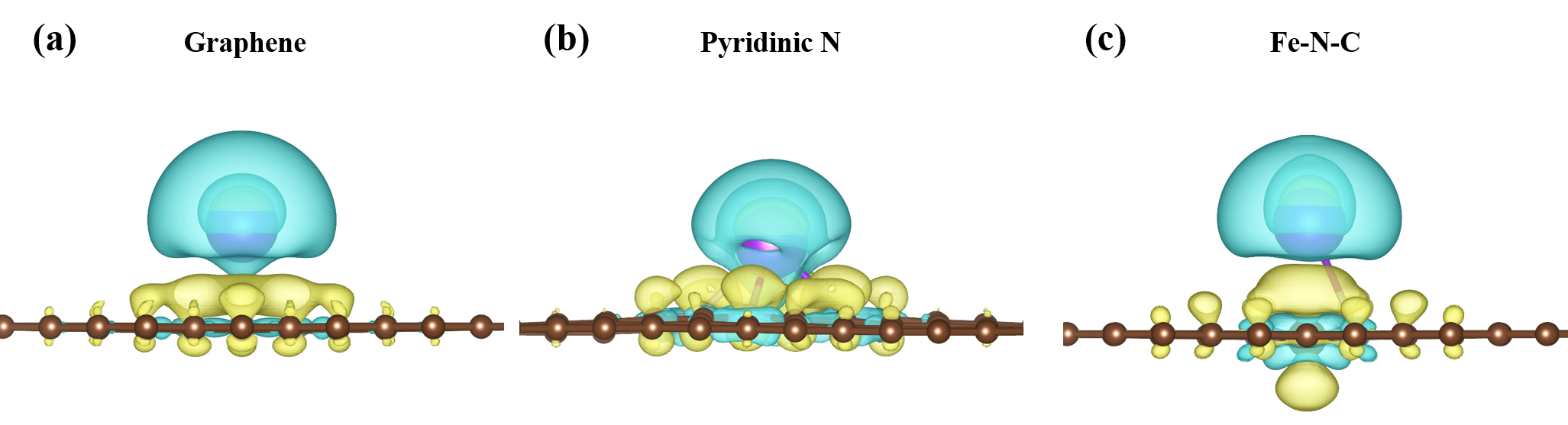


**Figure S20.** Side view of charge density difference with (a) Graphene, (b) Pyridinic N and (c) Fe-N-C.


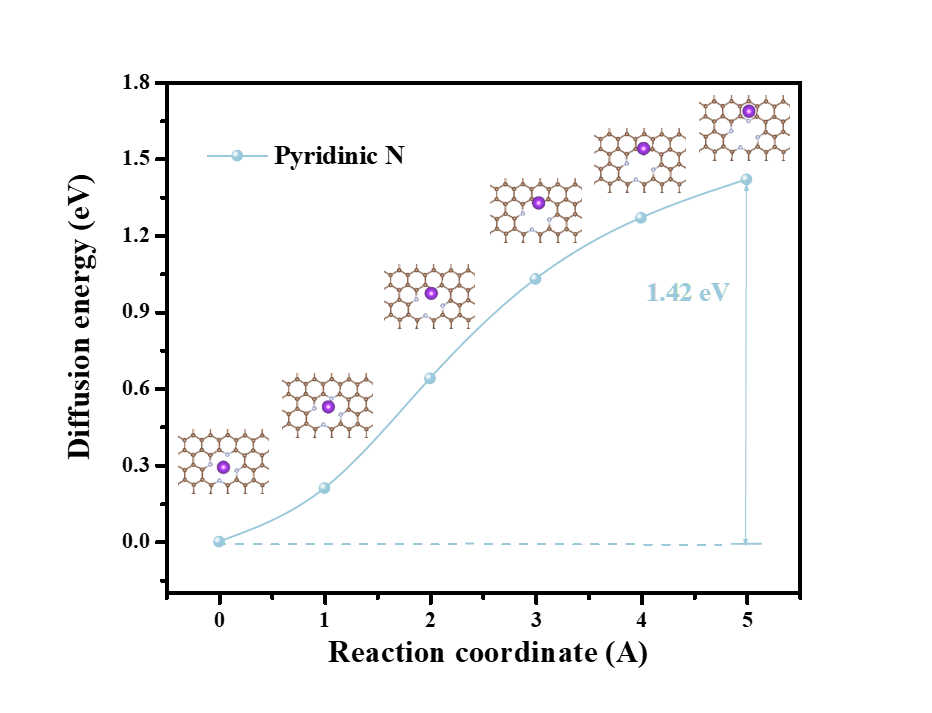


**Figure S21.** Migration path and energy barriers of K ion on Pyridinic N


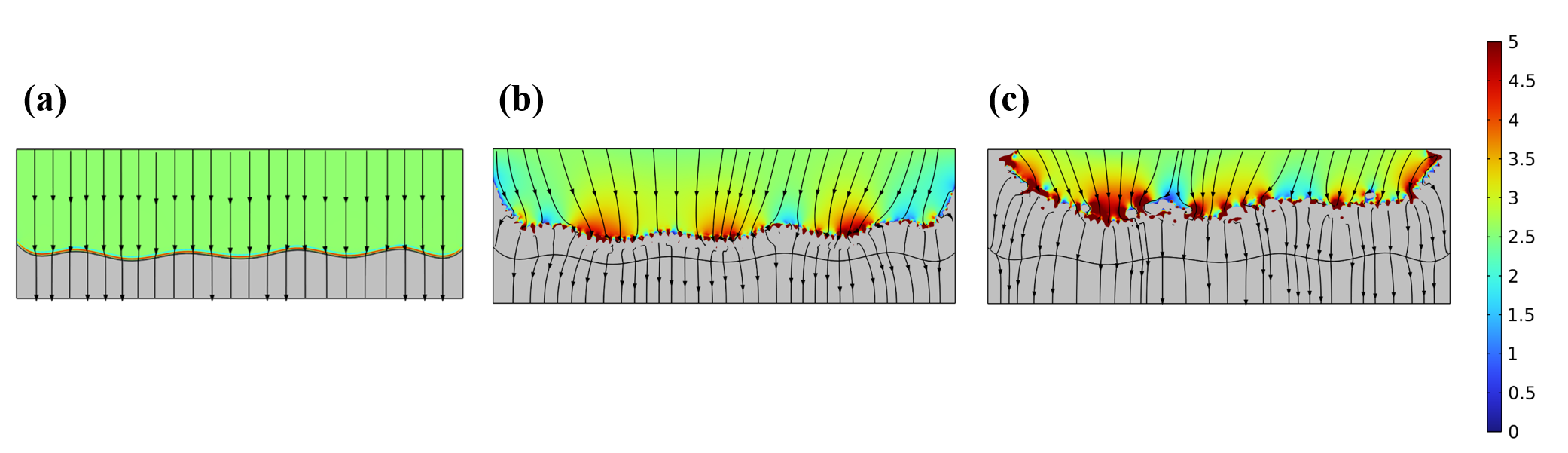


**Figure S22.** Current density distribution of CF at (a) initial state (b) intermediate state and (c) final state.


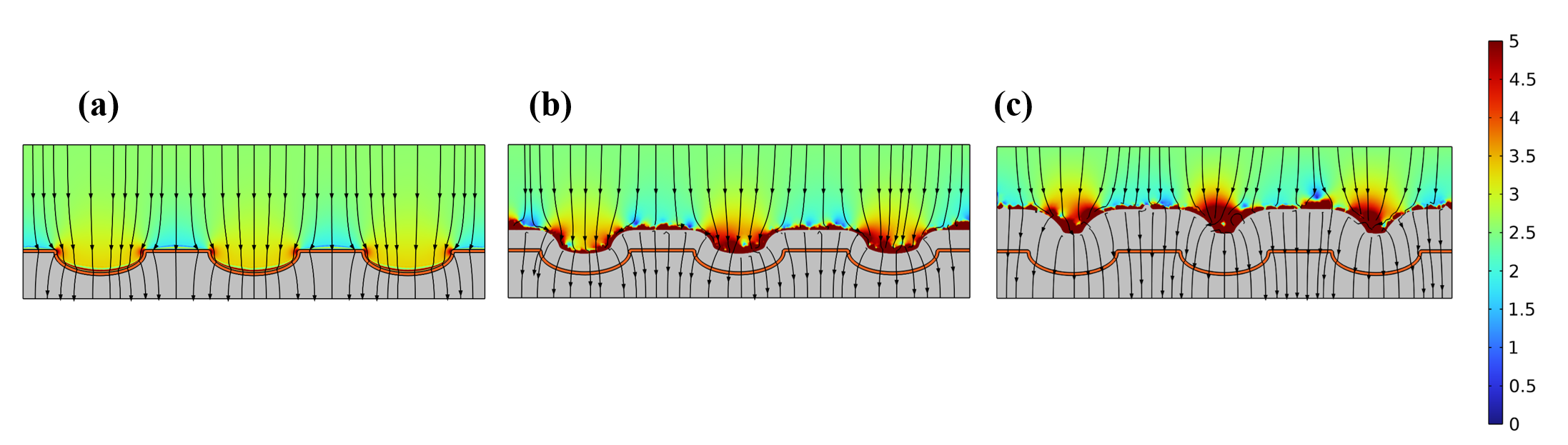


**Figure S23.** Current density distribution of Fe-N-PCF at (a) initial state (b) intermediate state and (c) final state.


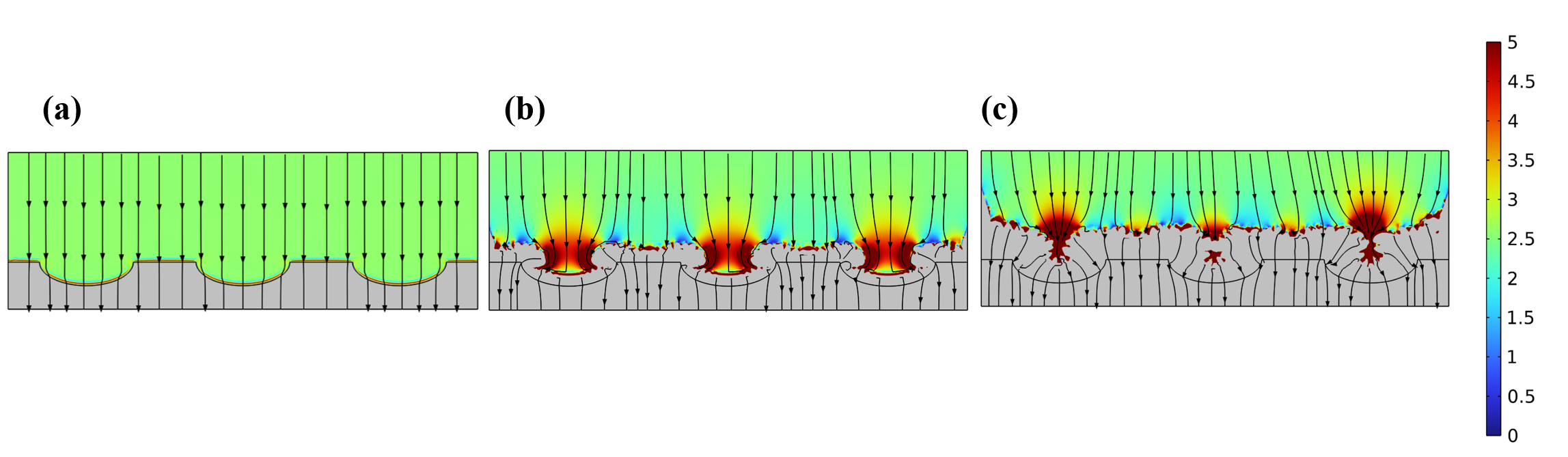


**Figure S24.** Current density distribution of N-PCF at (a) initial state (b) intermediate state and (c) final state.


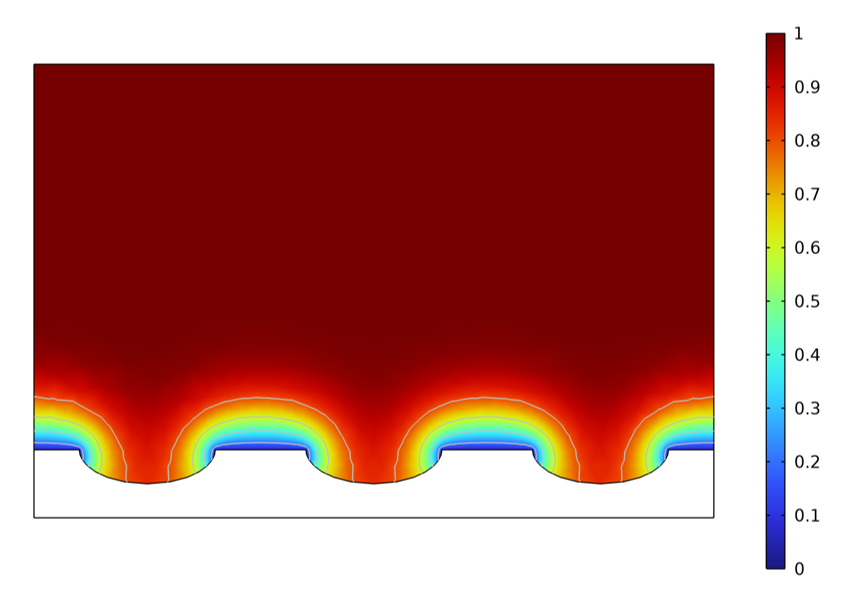


**Figure S25.** K ion flux distribution of N-PCF


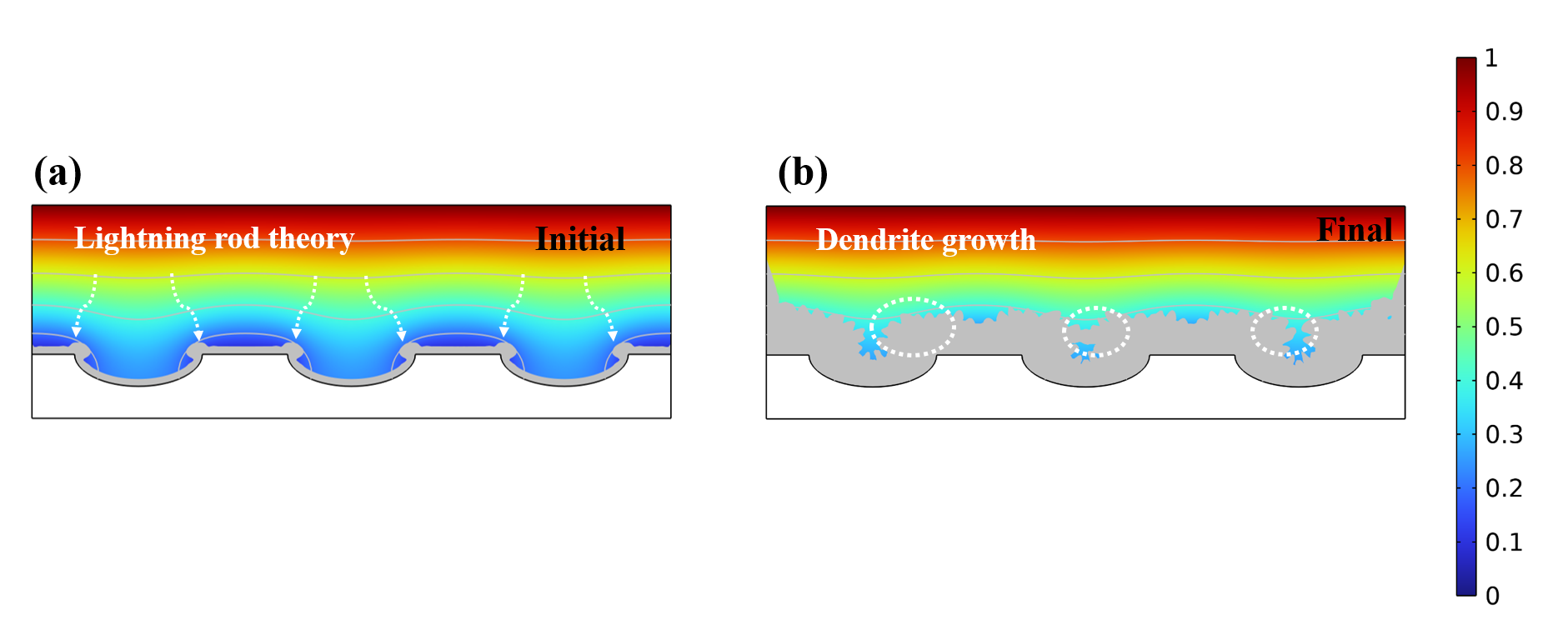


**Figure S26.** The morphology evolution snapshots during K deposition on N-PCF at (a) initial state and (b) final state


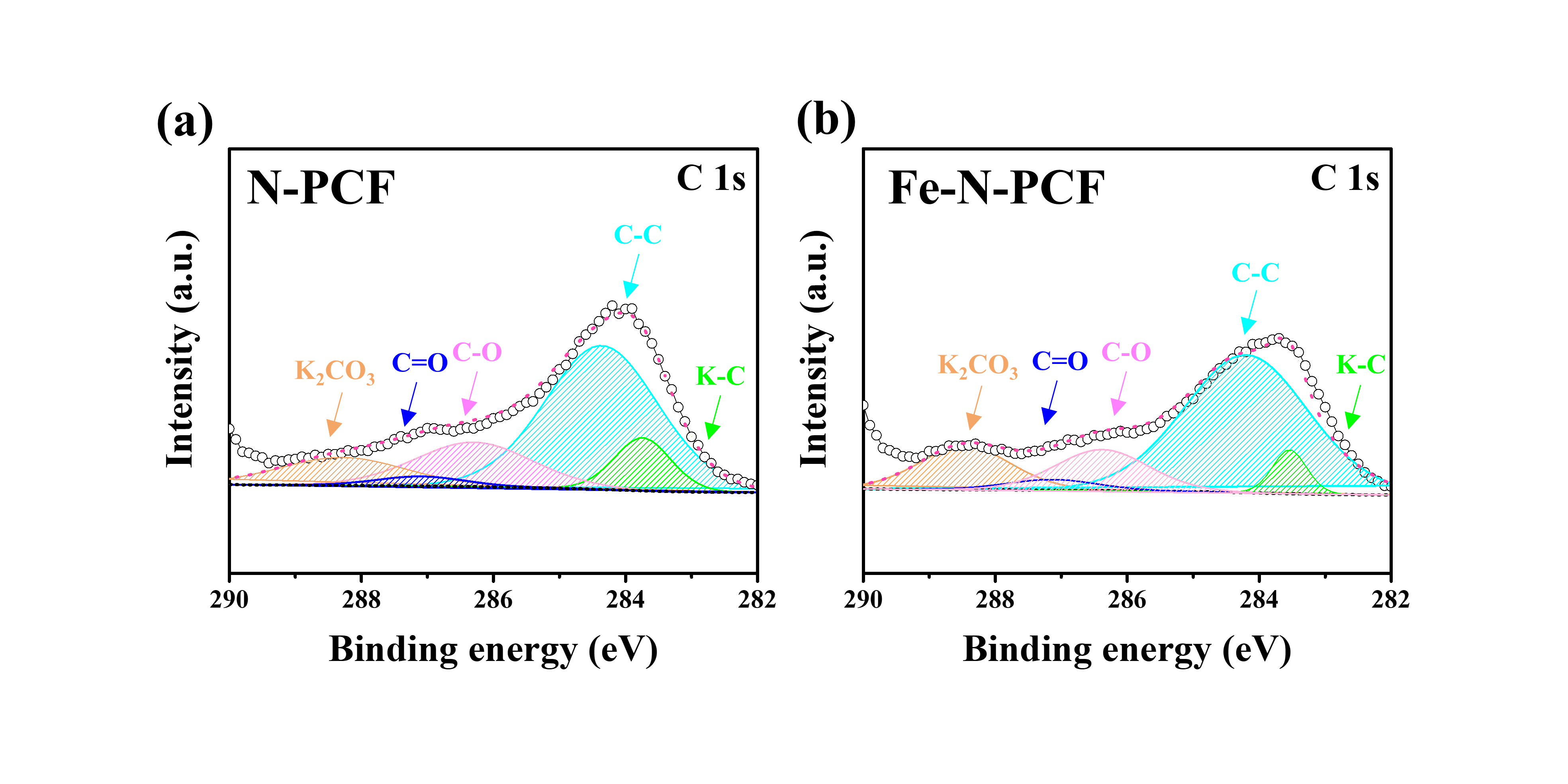


**Figure S27.** XPS profiles of C 1s spectra in N-PCF and Fe-N-PCF.

Table S1. Structural parameters extracted from the EXAFS fitting of Fe-N-PCF. (S_0_^2^=1)


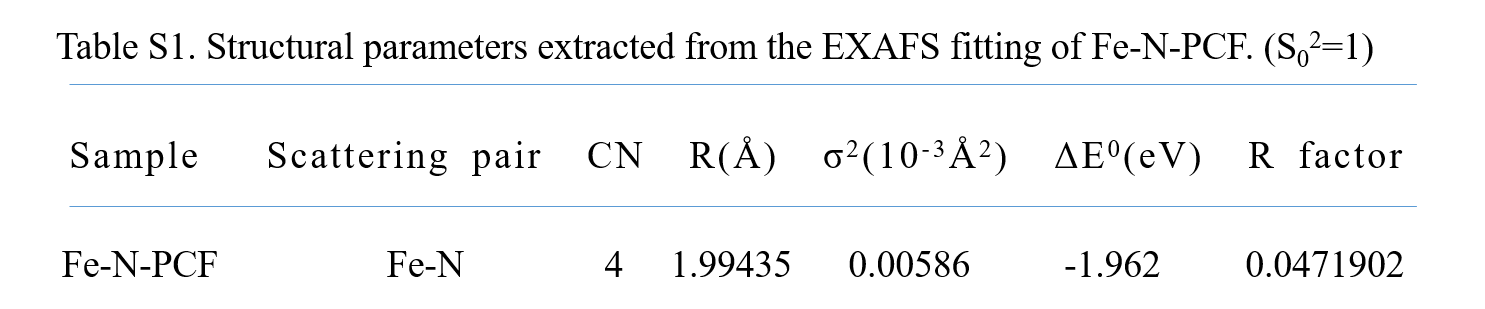


S_0_^2^ is the amplitude reduction factor; CN is the coordination number; R is the interatomic distance (the bond length between central atoms and surrounding coordination atoms), σ^2^ is Debye-Waller factor (a measure of thermal and static disorder in absorber-scatterer distances); ΔE^0^ is edge-energy shift (the difference between the zero kinetic energy value of the sample and that of the theoretical model). R factor is used to value the goodness of the fitting.

Table S2. Element contents of catalysts obtained from ICP of Fe-N-PCF.


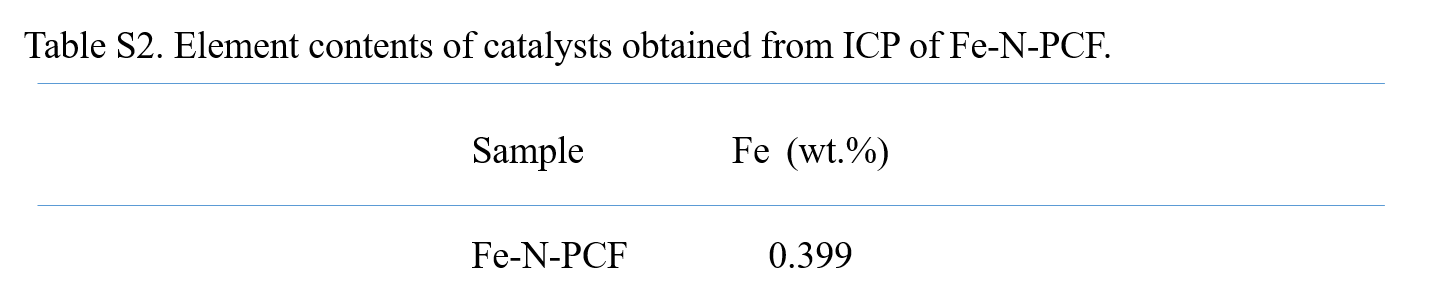


Table S3. The conductivity of N-PCF and Fe-N-PCF using four-point probe measurements.

|  | Ω/□ | Ω/□ | Ω/□ | Thickness (cm) | Ω*cm | S*m^-1^ |
| --- | --- | --- | --- | --- | --- | --- |
| **Fe-N-PCF** | 3112.1 | 2907.9 | 2894.5 | 0.0081 | 24.0691 | 4.15 |
| **N-PCF** | 2967.2 | 3026.2 | 3076.7 | 0.0092 | 27.8149 | 3.59 |

Table S4. Fitted EIS results of the different symmetric cells.

|  | Fe-N-PCF | N-PCF | PCF | K |
| --- | --- | --- | --- | --- |
| R_ct_ (Ω) | 1249 | 4982 | 5527 | 6874 |
